# Structure-Function Analysis of the Bifunctional CcsBA Heme Exporter and Cytochrome *c* Synthetase

**DOI:** 10.1128/mBio.02134-18

**Published:** 2018-12-18

**Authors:** Molly C. Sutherland, Nathan L. Tran, Dustin E. Tillman, Joshua M. Jarodsky, Jason Yuan, Robert G. Kranz

**Affiliations:** a Department of Biology, Washington University in St. Louis, St. Louis, Missouri, USA; University of Michigan—Ann Arbor

**Keywords:** CcsBA, ResBC, cytochrome *c*, cytochrome *c* biogenesis, heme, heme trafficking

## Abstract

Although intracellular heme trafficking must occur for heme protein assembly, only a few heme transporters have been unequivocally discovered and nothing is known about their structure or mechanisms. Cytochrome *c* biogenesis in prokaryotes requires the transport of heme from inside to outside for stereospecific attachment to cytochrome *c* via two thioether bonds (at CXXCH). The CcsBA integral membrane protein was shown to transport and attach heme (and thus is a cytochrome *c* synthetase), but the structure and mechanisms underlying these two activities are poorly understood. We employed a new cysteine/heme crosslinking tool that traps endogenous heme in heme binding sites. We combined these data with a comprehensive imidazole correction approach (for heme ligand interrogation) to map heme binding sites. Results illuminate the process of heme transfer through the membrane to an external binding site (called the WWD domain). Using meta-genomic data (GREMLIN) and Rosetta modeling programs, a structural model of the transmembrane (TM) regions in CcsBA were determined. The heme mapping data were then incorporated to model the TM heme binding site (with TM-His1 and TM-His2 as ligands) and the external heme binding WWD domain (with P-His1 and P-His2 as ligands). Other periplasmic structure/function studies facilitated modeling of the full CcsBA protein as a framework for understanding the mechanisms. Mechanisms are proposed for heme transport from TM-His to WWD/P-His and subsequent stereospecific attachment of heme. A ligand exchange of the P-His1 for histidine of CXXCH at the synthetase active site is suggested.

## Abstract (precis)

The movement or trafficking of heme is critical for cellular functions (e.g., oxygen transport and energy production); however, intracellular heme is tightly regulated due to its inherent cytotoxicity. These factors, combined with the transient nature of transport, have resulted in a lack of direct knowledge on the mechanisms of heme binding and trafficking. Here, we used the cytochrome *c* biogenesis system II pathway as a model to study heme trafficking. System II is composed of two integral membrane proteins (CcsBA) which function to transport heme across the membrane and stereospecifically position it for covalent attachment to apocytochrome *c*. We mapped two heme binding domains in CcsBA and suggest a path for heme trafficking. These data, in combination with metagenomic coevolution data, are used to determine a structural model of CcsBA, leading to increased understanding of the mechanisms for heme transport and the cytochrome *c* synthetase function of CcsBA.

## INTRODUCTION

Cytochrome *c* (referred to here as cyt *c*) functions in diverse electron transport chains to facilitate critical cellular functions, such as respiration and photosynthesis, and requires heme as a cofactor for proper folding and function (1–3). Cyt *c* is unique among cytochromes due to its requirement for covalently attached heme. The heme attachment reaction occurs away from the site of heme synthesis (4) outside the cytoplasm (prokaryotes), in the lumen (chloroplasts), or in the mitochondrial intermembrane space (eukaryotes) (3, 5–11). Thus, heme must be transported from inside (*n-*side/cytoplasm) to outside (*p-*side/periplasm) the cell and then be properly positioned for attachment via two thioether bonds to two cysteine (cys) thiols of a conserved CXXCH motif in apocyt *c*. The histidine of CXXCH then functions as one of the axial heme ligands in cyt *c*. Cyt *c* biogenesis can be accomplished by three pathways: system I (CcmABCDEFGH), system II (CcsBA), and system III (HCCS) (reviewed in references 3, 5, 6, 9, 10, 11, 12, and 13).

**FIG 1:**
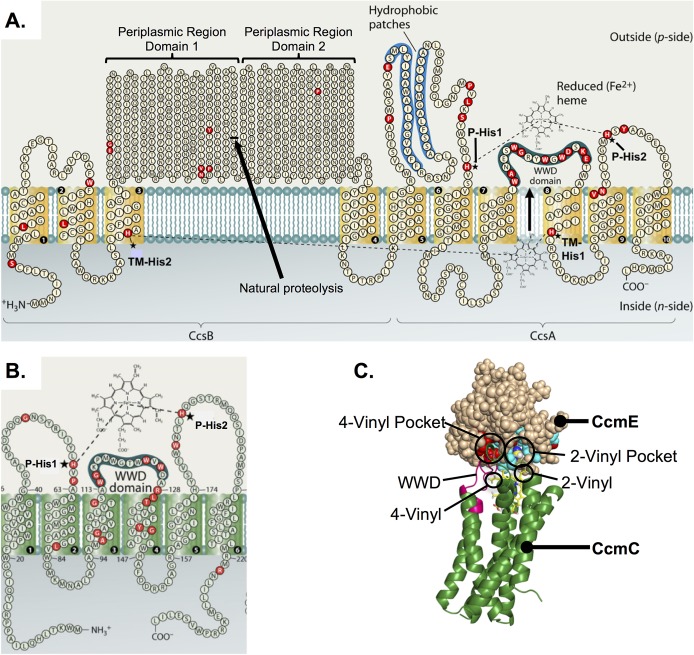
Comparison of CcmC and CcsBA. (A) Schematic of H. hepaticus CcsBA topology. CcsBA consists of 10 transmembrane domains and a large periplasmic region. The following conserved features are shown: two conserved histidines in the transmembrane domain (TM-His1 and TM-His2) and two conserved periplasmic histidines (P-His1 and P-His2) which flank the heme-handling WWD domain. The site of natural proteolysis at residue 368 is indicated with an arrow. Conserved residues as defined in reference 3 are indicated in red. (Panel A is modified from reference 3 with permission from the publisher.) (B) Schematic of E. coli CcmC topology. CcmC consists of six transmembrane domains. An external heme binding domain consists of the WWD domain and two conserved histidines (P-His1 and P-His2). (Panel B is modified from reference 3 with permission from the publisher.) (C) Model of CcmC-heme-CcmE interaction. A structural model of CcmC (green) was generated using metagenomic coevolution data and experimentally determined constraints to position heme in the WWD domain (magenta). This model was docked with a known structure of CcmE. (Panel C is modified from reference 30 with permission from the publisher.)

Here, we focus on the system II pathway, which consists of two integral membrane proteins, CcsB and CcsA, and was first identified by genetic analysis in *Chlamydomonas* (15–18), *Bacillus* (ResB and ResC) (19–21), and *Bordetella* (22, 23). A subset of system II pathways, such as those found in *Helicobacter*, *Bacteroides*, *Wolinella,* and *Campylobacter*, encode CcsBA as a single, fused open reading frame (ORF) (3, 24). Recombinant expression of the Helicobacter pylori (25) and H. hepaticus (24) CcsBAs in Escherichia coli demonstrated that CcsBA is sufficient for cyt *c* maturation and therefore is the holocyt *c* synthetase able to replace the functions of all eight system I genes (*ccmA* to *ccmH* [*ccmA*-*H*]) (24, 25). Conserved features of CcsBA include a large periplasmic region in CcsB, a tryptophan-rich WWD domain in CcsA, and four conserved histidine residues (one in the transmembrane [TM] of CcsB, one in the TM of CcsA, and two in the CcsA periplasmic domain [see Fig. 1A]) (14, 18, 24, 26, 27). Recombinant expression of H. hepaticus CcsBA allowed the first biochemical purification of CcsBA and suggested that CcsBA is a bifunctional enzyme that both transports heme across the bacterial inner membrane and attaches it to cyt *c* (24). However, while recombinant H. hepaticus CcsBA is functional *in vivo*, purified CcsBA protein is proteolyzed in the middle of the large periplasmic region (24), thus making unclear whether it is active. Here we engineered homogenous preparations of CcsBA by insertion of stop/start cassettes at defined junctions, including the proteolysis site, with results indicating that the “proteolyzed” protein retains function. Deletion analysis in this large periplasmic region (535 residues) also suggests the presence of two separate domains, each of approximately 250 residues.

Previously, mutagenesis of the four histidines and triple mutation of the WWD domain (WWXD→AAXA) revealed that all are required for cyt *c* maturation (14, 24, 27) and the TM-His were shown to function as axial ligands for heme (24, 27). However, a role of heme binding by the WWD domain has not been demonstrated for CcsBA and nothing is known about its structural features. Here we prove the WWD domain binds endogenous heme by the use of a new cysteine/heme crosslinking approach and show that the P-His function as axial heme ligands. Recently introduced structural programs that utilize coevolution data, combined with the WWD heme crosslinking data, were employed to propose the structural basis for heme transport and the cyt *c* synthetase activity.

## RESULTS

### Structural convergence of the heme-handling protein family: CcsBA and CcmC.

The heme-handling membrane protein family consists of CcsBA, CcmC, and CcmF (28, 29) and is defined by two conserved periplasmic histidines (P-His1 and P-His2) flanking a tryptophan-rich region (WWD domain) that was previously proposed to interact with heme (29) (Fig. 1A and B). Recent structure-function analysis of CcmC suggested that the WWD domain directly and stereospecifically positions heme for subsequent attachment to CcmE (30, 31). Binding of CcmE (the acceptor protein) to CcmC is required for access of heme to the WWD domain (Fig. 1C) (32). However, in contrast to the CcsBA protein, the mechanism by which heme moves to the CcmC WWD domain remains unknown.

**FIG 2:**
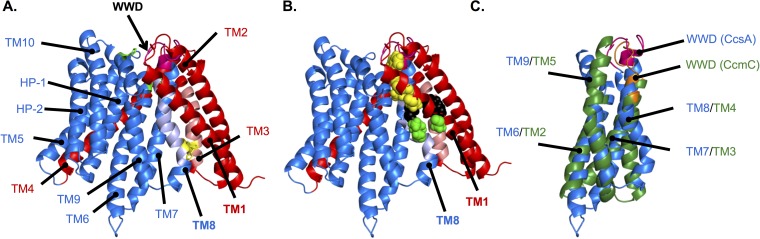
Molecular modeling of CcsBA. (A) Rosetta *de novo* modeling constrained by genomic coevolution data was used to predict the structure of CcsBA (lacking the periplasmic region (aa 98 to 633)). CcsB is red, CcsA is blue, and the WWD domain is magenta. TMs are labeled. The TM-His (yellow) in TM3 (salmon) and TM8 (light blue) and P-His residues (green) have not yet been modeled to form heme binding sites. (B) Genomic coevolution data suggest that CcsB TM1 and CcsA TM8 interact. Examples of coevolved pairs are shown as follows: G27/W845 (yellow), T30/W845 (yellow), Y22/G651 (black), and G20/I655 (green). (C) Overlay of predicted CcmC TM2 to TM5 (green) and CcsA TM6 to TM9 (blue) form a core region around the conserved WWD domain. Note that in this overlay, the WWD domains have not yet been modeled to form heme binding sites (see Fig. 6B).

A major gap in the cyt *c* biogenesis field has been the lack of a structural framework for understanding the function of WWD containing proteins. The method recently developed by the Baker group using metagenomic sequences in structural predictions has been successfully applied to membrane proteins (33, 34). The system I and II proteins are particularly suited for this approach due to their deep evolution and large number of homologs. Experimentally determined constraints and genomic coevolution data were recently used to model a structure of CcmC-heme-CcmE (30) (Fig. 1C). While CcmC and CcsBA both contain the conserved WWD domain flanked by P-His1 and P-His2, CcsBA is much larger, with additional features that include two TM histidines (TM-His1 and TM-His2) and a large periplasmic region (residues 98 to 633) (Fig. 1A). To gain insights into CcsBA, a structural model of CcsBA was generated using genomic coevolution data (GREMLIN) and the Rosetta modeling suite, resulting in convergence of the TM regions over the top 10 models (Fig. 2A; see also Fig. S1 in the supplemental material). Initially, genomic coevolution analysis (Jackmmer, GREMLIN) of CcsBA did not generate sufficient sequences for analysis due in part to the largely unconserved periplasmic region of CcsBA (amino acids [aa] 98 to 633). CcsBA lacking the periplasmic region (aa 1 to 94 and 634 to 936) could be modeled (see also Text S1 in the supplemental material). The Rosetta *ab initio* protocol predicted that hydrophobic patch 1 (HP-1) and HP-2 (Fig. 1A) are transmembrane domains (Fig. 2A). Because HP-1 and HP-2 do not form part of the core (see below) and because there have been two published PhoA fusion studies (18, 26) that placed them in the periplasm, we have provisionally retained their designation as HP rather than TMs. There are many CcsB TM residues that are predicted to interact with residues in CcsA. For example, genomic coevolution data indicate that TM1 and TM8 interact (Fig. 2B). Importantly, the four transmembrane domains immediately flanking the WWD in CcmC and CcsBA are predicted to have similar core structures (Fig. 2C), suggesting conservation within the heme-handling protein family at the levels of both structure and function (heme delivery). One caveat for GREMLIN- and Rosetta-based structural prediction is that cofactors and ligands are not modeled, resulting in models that can obscure their binding sites (33). Thus, we wanted to examine the function and properties of these sites using various approaches, with this experimental information facilitating further structural prediction of CcsBA.

10.1128/mBio.02134-18.1 TEXT S1 Supplemental Materials and Methods. Download Text S1, PDF file, 0.1 MB. Copyright © 2018 Sutherland et al. 2018 Sutherland et al. This content is distributed under the terms of the Creative Commons Attribution 4.0 International license.

10.1128/mBio.02134-18.2 FIG S1 Ensemble of CcsBA modeling. (A) Side view of an overlay of the top 10 models of CcsBA. The periplasmic region (residues 99 to 633) were removed for Gremlin and Rosetta modeling. Each transmembrane domain (TM) and hydrophobic patch (HP) is individually colored. (B) Top view of an overlay of the top 10 models of CcsBA. CcsA is blue, CcsB is red, the WWD domain is magenta, and the TM-His are shown in yellow. No modeling of heme binding sites has been performed in these models. Download FIG S1, JPG file, 1.0 MB. Copyright © 2018 Sutherland et al. 2018 Sutherland et al. This content is distributed under the terms of the Creative Commons Attribution 4.0 International license.

### The transmembrane histidines are required for heme transport to the external heme binding domain: a different role for P-His1 than for P-His2.

**FIG 3:**
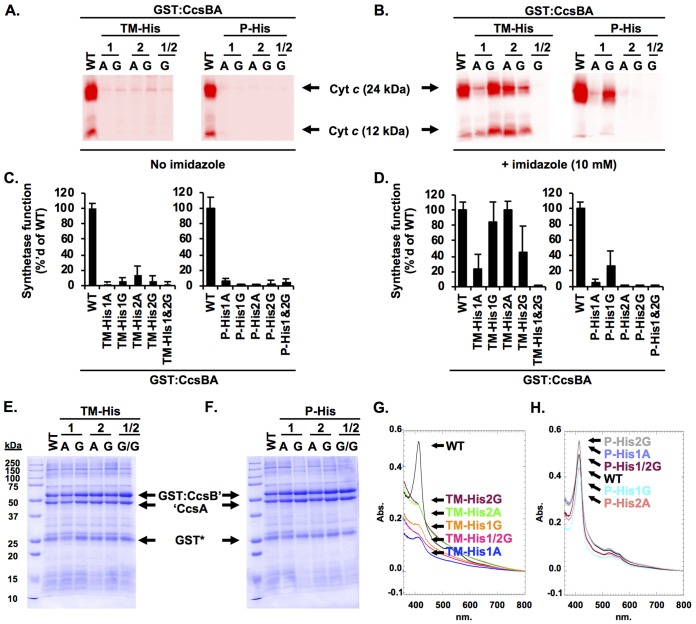
Conserved histidines are required for CcsBA function and heme ligand formation. (A) The CcsBA His variants were coexpressed with cyt *c*_4_ in E. coli Δ*ccm*. The ability of variants to mature cyt *c*_4_ was monitored by cell lysis, separation by SDS-PAGE, and heme staining. WT, wild type. (B) Chemical complementation of CcsBA His variants with imidazole. Strains were supplemented with 10 mM imidazole during growth, and cyt *c*_4_ maturation was monitored as described above. (C and D) Quantitation of the data in panel A and B with the wild-type levels normalized to 100%. Data represent results from three triplicate experiments. (E and, F) Variants were subjected to affinity purification using a GST tag, and 10-µg volumes were separated via SDS-PAGE and visualized with Coomassie total protein stain. (G and H) Quantification of heme copurification via UV-vis Soret (410 nm) with 100 µg of protein. Abs, absorbance.

CcsBA contains four conserved histidines (14, 18, 24, 27, 35), including two histidines in the transmembrane domains (TM-His1 and TM-His2) and two in the periplasmic space (P-His1 and P-His2) flanking the conserved WWD domain (see Fig. 1A). The P-His residues are named based on homology to system I proteins, CcmF and CcmC (10, 36). Initial characterization of the conserved histidines was previously undertaken using the traditional approach of mutation to alanine. The results demonstrated that each histidine was required for heme attachment to cyt *c* (14, 18, 24, 27) (Fig. 3A and C). The TM-His were additionally shown to function as likely heme ligands by chemical complementation with exogenous imidazole of the TM-His→Ala variants for heme attachment (24, 27) (Fig. 3B and D). Imidazole is the histidine side chain, and when a cavity is created by mutating a histidine residue, imidazole can sometimes enter the cavity, restoring heme ligand formation and protein function, as demonstrated in a recombinant form of myoglobin with a His93Gly mutation (37). Crystallization of this mutant revealed imidazole in the His93Gly cavity and bonded to the heme iron (37); thus, chemical correction of a histidine mutation by exogenous imidazole is used as evidence for axial heme ligand involvement. Unfortunately, the P-His→Ala variants were not chemically complemented in either H. hepaticus (24) (Fig. 3B and D) or Wolinella succinogenes CcsA2 (which recognizes CXXCH) (27). One explanation could be that the alanine side chain results in a smaller cavity (compared to that seen with glycine) that may not accommodate the exogenous imidazole. To test this, each conserved histidine was mutated to glycine. All variants were assayed for heme attachment function by coexpression with cyt *c*_4_ in E. coli lacking the endogenous cyt *c* biogenesis genes (E. coli Δ*ccm*). Similarly to the His→Ala variants, the His→Gly variants were not functional for heme attachment to cyt *c* (Fig. 3A and C). Using the imidazole correction assay, variants were used to assess the role of the conserved histidines as heme ligands. The TM-His→Gly and P-His→Gly variants were coexpressed with cyt *c*_4_ in E. coli Δ*ccm* and grown in the presence of 10 mM imidazole. Both the TM-His→Ala and TM-His→Gly variants were chemically complemented by exogenous imidazole for cyt *c* maturation (Fig. 3B and D). The TM-His1Gly/TM-His2Gly double variant was not, possibly suggesting that imidazole is retained only transiently in the histidine cavity. P-His1Gly, but not P-His2Gly, was chemically complemented with imidazole (Fig. 3B and D), providing evidence that the P-His1 can function as a heme ligand. The data also suggest that P-His1 and P-His2 are not equivalent with respect to their function. In the Discussion, the structure of this external heme binding domain is used to propose a mechanism involving a different (or additional) role for P-His1 in comparison to P-His2.

To extend the structure-function analysis of the conserved histidines, a biochemical analysis was carried out. CcsBA variants were subjected to glutathione *S*-transferase (GST) affinity purification and shown to be stable, with stoichiometric GST:CcsB' and 'CcsA polypeptides in the preparations (Fig. 3E and F). Copurification of heme was assessed using the Soret region (410 nm) of the UV-visible light (UV-vis) spectra (Fig. 3G and H). Similarly to analysis of the TM-His and P-His alanine variants (24), mutation of the TM-His→Gly resulted in ∼15% of copurified heme compared to the wild type (Fig. 3G). In contrast, the P-His→Gly variants copurified with wild-type levels of heme (Fig. 3H). UV-vis spectral analysis of the glycine variants revealed characteristic alpha (∼560) and beta (∼531) peaks in the sodium dithionite reduction spectra (Fig. S2). Pyridine hemochrome assays determined that the His→Gly variants copurify with *b*-type heme in a manner similar to that seen with the wild type (Fig. S2, insets). These data are consistent with the previously published proposal (24) that the TM-His residues form an initial heme binding site in the transmembrane domains prior to heme entering the external heme binding site.

10.1128/mBio.02134-18.3 FIG S2 UV-visible spectral analysis of conserved His→Gly variants. The four conserved histidine residues were mutated to Gly. Results of UV-vis spectroscopy of 100 μg of purified protein are shown as follows: as purified (black), after sodium dithionite reduction (red), and pyridine hemochrome indicative of *b*-type heme (inset, green). Relevant peaks are labeled. Download FIG S2, JPG file, 2.4 MB. Copyright © 2018 Sutherland et al. 2018 Sutherland et al. This content is distributed under the terms of the Creative Commons Attribution 4.0 International license.

It is likely that the reduced levels of heme and disruption of heme transport are responsible for the TM-His→Gly heme attachment defect (Fig. 3A and C). In contrast, the P-His→Gly variants retain wild-type levels of heme. In principle, the heme in wild-type CcsBA could be in the external heme domain (P-His) or in both the TM-His and external heme binding domains, while all of the heme could reside in the transmembrane heme binding domain in the P-His mutants. One possible approach to test this is to determine the heme redox potentials of wild-type CcsBA, P-His1Gly, and P-His2Gly in the hope that the different redox potentials could be used to clarify the location and environment of heme. The redox potential of wild-type CcsBA was −115 mV (see Table S1 in the supplemental material), while P-His1Gly and P-His2Gly had redox potentials of −119 mV and −122 mV, respectively (Table S1), indicating that the chemical environments of heme are similar. Although the redox potential data will be valuable in analyzing future synthetase mechanisms, it remained necessary to test where heme is located in wild-type CcsBA and to structurally map heme in these domains.

10.1128/mBio.02134-18.7 TABLE S1 CcsBA heme redox potentials. Download Table S1, PDF file, 0.05 MB. Copyright © 2018 Sutherland et al. 2018 Sutherland et al. This content is distributed under the terms of the Creative Commons Attribution 4.0 International license.

### Structure-function analysis of the WWD domain by cysteine/heme crosslinking: heme binds independently of the apocytochrome *c* substrate.

**FIG 4:**
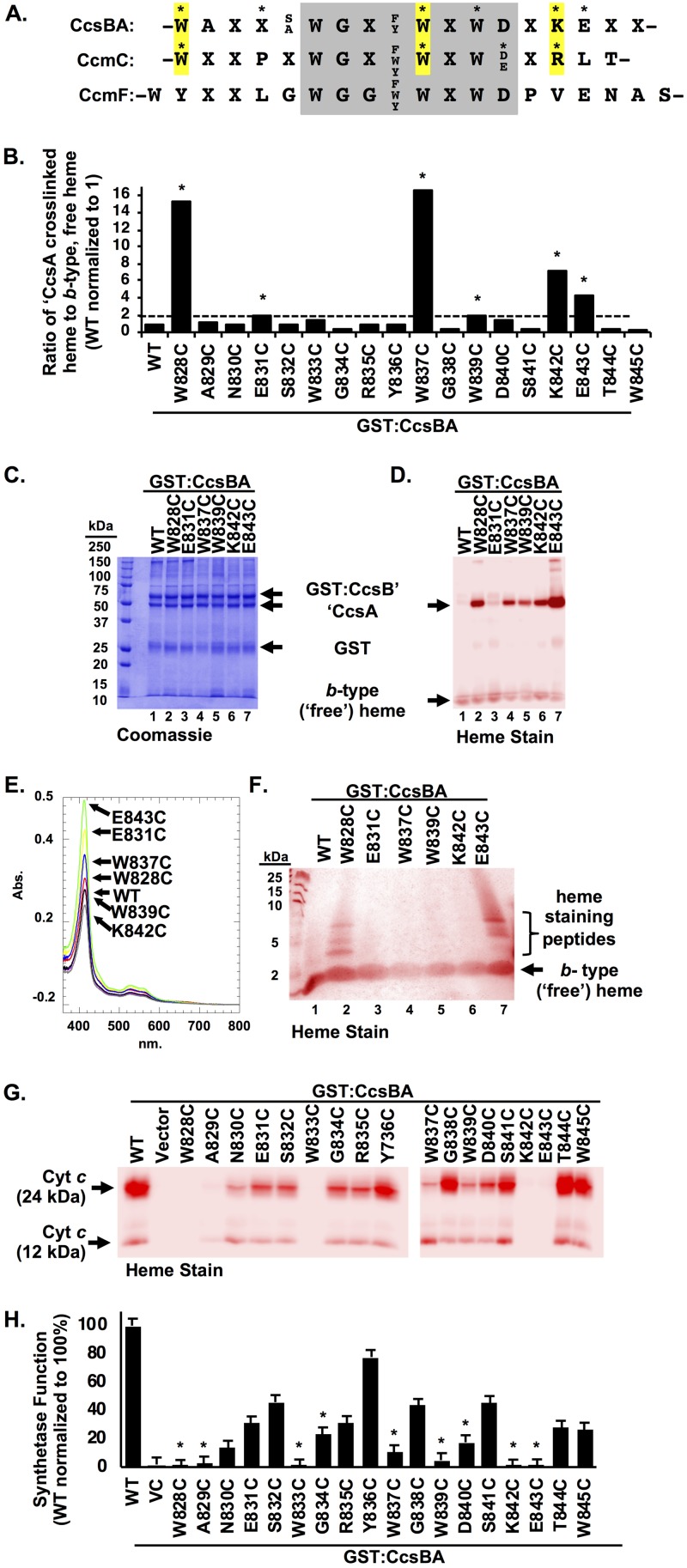
CcsBA WWD domain cysteine/heme crosslinking. (A) Sequence alignment of the following WWD domains from the prokaryotic cyt *c* biogenesis pathways: CcsBA (system II), CcmC (system I), and CcmF (system I). Invariant residues and semiconserved residues are indicated, and variable residues are indicated with an “X.” CcsBA and CcmC residues that form a cys/heme crosslink are indicated with an asterisk. Homologous residues of CcsBA and CcmC that crosslink are highlighted in yellow. (B) Each residue of the WWD domain was individually mutated to Cys. GST:CcsBA Cys variants were subjected to affinity purification and assayed for an increase in the heme ratio of ′CcsA-associated heme to *b*-type (“free”) heme. Variants with ≥2× the ratio of wild type were selected for further analysis (asterisks). (C and D) Affinity purification (C) and heme staining (D) of selected variants. Data are representative of three purifications. (E) Heme copurification was monitored via heme Soret (410 nm). Data are representative of three independent purifications. (F) Confirmation of covalent crosslinking to heme via the cysteine residue was performed by trypsin digestion of purified protein, separation via Tris-tricine SDS-PAGE, and heme staining. Crosslinked heme is retained on trypsinized peptides. (G) Function of GST:CcsBA variants shown by coexpression with cyt *c*_4_ in E. coli Δ*ccm* and monitoring of the ability to mature cyt *c* via cell lysis, SDS-PAGE, and heme staining. (H) Quantification of data determined as described for panel G. Asterisks indicate conserved residues. Three independent triplicate experiments were performed.

The conserved WWD domain is a tryptophan-rich domain found in CcsBA, as well as in the system I proteins CcmC and CcmF (Fig. 4A), that is proposed to interact with heme (14, 26, 28, 29). It was hypothesized that in all three members (CcsA, CcmC, and CcmF), the two P-His function as axial ligands to the heme whereas the conserved WWD domain binds and positions heme for attachment to the acceptor protein (CcmE for CcmC and CXXCH of cyt *c* for CcsA and CcmF). A novel cysteine/heme crosslinking approach that exploits the natural propensity of a cysteine thiol and heme vinyl group to form a covalent bond (i.e., crosslink) when in close proximity (Fig. S3A) was recently used to map heme in the CcmC WWD domain (30). To analyze the CcsBA WWD domain and test the utility of the cys/heme crosslinking approach, a comprehensive panel of cysteine substitutions were engineered into the 18 residues of the WWD domain (Fig. 4A and B). To determine if the CcsBA WWD domain directly interacts with heme, the Cys variants were subjected to affinity purification using GST (Fig. S3B), and upon SDS-PAGE analysis, the ratio of 'CcsA-bound heme to *b*-type (“free”) heme was determined (Fig. 4B; see also Fig. S3C). Note that copurified *b*-type heme runs at the dye front as free heme upon separation by SDS-PAGE (for an example, see Fig. 4D). Six cysteine variants that had greater than 2× the wild-type 'CcsA to *b*-type heme ratio (Fig. 4B, asterisks) were selected for further analysis, shown to be stable (Fig. 4C), and purified with increased heme at the 'CcsA polypeptide (Fig. 4D).

10.1128/mBio.02134-18.4 FIG S3 Cysteine/heme crosslinking in the conserved WWD domain of CcsBA to test for heme presence and mapping. The 18 residues of the WWD domain were each mutated to cysteine and purified using an N-terminal GST tag. (A) Schematic of cysteine/heme crosslink reaction. A single cysteine thiol is shown for simplicity, and red arrows represent a 2-electron transfer. (Panel A is modified from reference 30 with permission.) (B and C) Purified proteins were assessed for stability (Coomassie) (B) and heme association with CcsA′ (heme stain) (C). (D to J) UV-vis spectroscopy of purified variants, as purified (black) and reduced (red). (K) Pyridine hemochrome spectra with variants labeled. (L) Quantification of heme levels based on data from the Soret region (410 nm). Three independent purifications were performed. (M) Purified variants were subjected to limited trypsin proteolysis, separated by tricine SDS-PAGE, and silver stained to determine the extent of proteolysis. Download FIG S3, JPG file, 2.3 MB. Copyright © 2018 Sutherland et al. 2018 Sutherland et al. This content is distributed under the terms of the Creative Commons Attribution 4.0 International license.

In the case of CcmC, heme was found in the WWD domain only when the acceptor CcmE was present (30). In contrast, heme can be crosslinked to the CcsBA WWD domain in the absence of the acceptor (apocyt *c*). The amount of copurified heme was determined by the absorbance value determined for the Soret region, 410 nm (Fig. 4E; see also Fig. S3L), demonstrating that all variants copurified with approximately wild-type levels of heme. The purified CcsBA Cys variants have UV-visible spectra similar to those determined for wild-type CcsBA (Fig. S3D to J). Pyridine hemochrome spectrum analyses were performed to determine if a covalent cys/heme crosslink was formed; however, all variants exhibited an alpha peak of 556 nm (Fig. S3K), indicating that the majority of heme in the protein complexes is *b*-heme. This suggests that a significant amount of heme may be retained in the transmembrane domain (i.e., liganded by TM-His1 and TM-His2) or, alternatively, that only some of the heme present in the WWD domain is crosslinked. Therefore, an alternative strategy to verify covalent cys/heme crosslinking was undertaken. CcsBA complexes were subjected to limited trypsin proteolysis with the assumption that covalently bound heme would be retained on trypsin peptides whereas *b-*heme would migrate as free heme on a tricine SDS-PAGE gel. Of the six CcsBA variants, two (W828C and E843C) retained detectable heme on trypsin peptides (Fig. 4F), demonstrating that a cys/heme crosslink had formed. All partial trypsin digestions produced trypsin peptides (Fig. S3M). Because W828C and E843C contain the most crosslinked heme (Fig. 4D, lanes 2 and 7), the other variants were likely below detection limits of the heme-stained tricine gel. These results demonstrate that the CcsBA WWD domain directly binds heme and, together with the P-His axial ligands, comprises the external heme binding domain. Although some CcsBA-associated heme likely resides in the TM-His heme domain, heme must be transferred and bound by the external heme binding domain prior to attachment to cyt *c*.

### The conserved WWD domain is required for CcsBA synthetase function.

Next, the role of the WWD domain in heme attachment was examined. Previously, mutation of the WWXD motif to AAXA in H. hepaticus CcsBA, as well as W. succinogenes system II proteins CcsA1 and CcsA2, prevented heme attachment to apocyt *c* (24, 27). In the plastid CcsA encoded system II protein of Chlamydomonas reinhardtii, single mutations of Trp288→Ala (AXWD) and Trp290→Ala (WXAD) of the WXWD motif resulted in reduced heme attachment function (<20%), while an Asp291→Ala mutation of the WXWD motif did not affect heme attachment to soluble cyt *c*6 (14). A double mutation (WXSD→AXAD) was nonfunctional (14). These results demonstrate that this conserved motif is required for heme attachment in system II pathways; however, a comprehensive genetic analysis of the WWD domain and the 18 residues that comprise it had not been undertaken.

Each CcsBA Cys substitution in the WWD domain was tested for the ability to attach heme to cyt *c* to ascertain cyt *c* synthetase activity. The WWD Cys variants were coexpressed with cyt *c*_4_ in E. coli Δ*ccm*, and levels of cyt *c* maturation were assessed (Fig. 4G and H). Five residues (W828, A829, W833, K842, and E843) were required for CcsBA function, and five substitutions (N830, G834, W837, W839, and D840) reduced CcsBA function to below 25% of the wild-type level. Seven substitutions (E831, S832, R835, G838, S841, T844, and W845) reduced CcsBA function to between 25% and 50% of the wild-type level, and one residue (Y836) retained ∼80% of the wild-type function (Fig. 4G and H). Not surprisingly, this analysis showed that conserved residues (Fig. 4H, asterisks) are required for CcsBA synthetase function and that partially or nonconserved residues are less critical for synthetase function.

**FIG 5:**
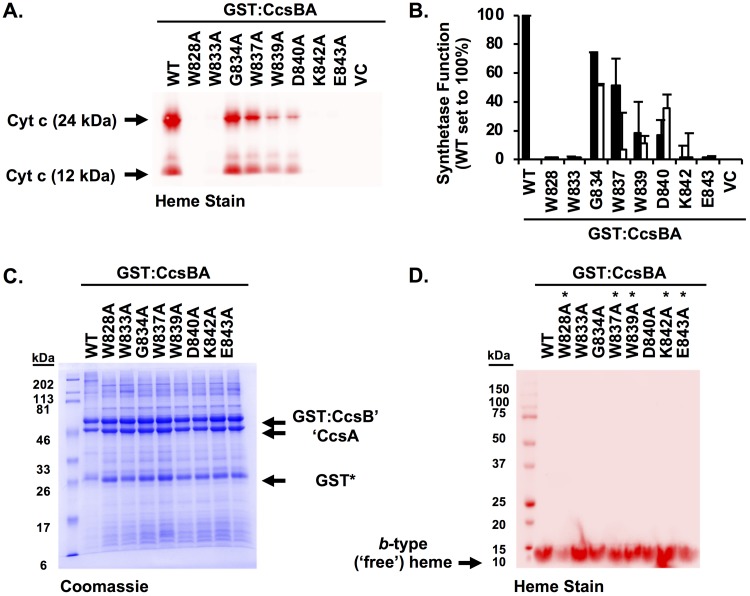
Alanine scanning of the conserved CcsBA WWD domain. (A) The CcsBA WWD Ala variants were coexpressed with cyt *c*_4_ in E. coli Δ*ccm*. Cells were lysed, separated by SDS-PAGE, and analyzed by heme staining to determine the ability to attach heme to cyt *c*. (B) Quantitation of the data determined as described for panel A, where the wild-type level is normalized to 100%. Black bars represent Ala variants; Cys variants (data from Fig. 4H) are shown as white bars. Data represent results from three triplicate experiments. (C and D) GST affinity purification of CcsBA variants were assessed for stability by Coomassie total protein staining (C) and heme staining (D). Asterisks denote the five highest cys/heme crosslink substitutions, confirming that the cysteine (thiol) is required for heme crosslinking.

To extend the genetic analysis of the WWD domain and to ascertain if the observed defects in synthetase function were due to the particular Cys substitutions, most conserved residues were mutated to alanine (Fig. 5A). CcsBA Ala variants were tested for the ability to attach heme to cyt *c* (Fig. 5A and B), with the wild-type level normalized to 100%. Four residues with Ala substitutions (W828, W833, K842, and E843) were required for CcsBA function, two residues (W839 and D840) reduced CcsBA function to below 25%, and two residues (G834 and W837) retained greater than 50% of the wild-type level of synthetase function (Fig. 5A and B, black bars). Substitution of residues to Ala or Cys resulted in similar levels of CcsBA synthetase function (Fig. 5A and B; compare black bars to white bars). Residues homologous to W828 and W833 were not functional in C. reinhardtii when mutated to Ala (12), suggesting that the function of this domain is conserved across system II pathways, whether in separate *ccsA* ORFs or in the fused *ccsBA* ORFs.

To gain further insight into the structure and function of the WWD domain, the Ala substitutions were biochemically characterized. GST affinity purifications demonstrated that alteration of the WWD domain did not affect protein stability (Fig. 5C). UV-vis spectral characterization of WWD Ala variants resulted in wild-type spectra (Fig. S4). Pyridine hemochrome assays revealed that the WWD Ala variants copurified with *b*-type heme at wild-type levels (Fig. S4, insets). The heme redox potential of W828A was approximately −125 mV (Table S1), similar to the wild-type level. To determine if the observed cys/heme crosslinking was specific to the cysteine mutation, alanine substitutions at the same WWD residues were analyzed for heme crosslinks (Fig. 5D). None of the alanine substitutions showed increased heme levels in CcsA (Fig. 5D) as the cysteine substitutions did (Fig. 4D). This suggests that specific bonds of thioether to heme vinyl represent the basis for the cys/heme crosslinks. We discuss below a striking correlation between the cysteine substitutions in the CcsA and CcmC WWD domains that crosslinked to heme vinyl groups (see yellow highlighted residues in Fig. 4A) and use information from the CcmC structural mapping to model the CcsA external heme domain.

10.1128/mBio.02134-18.5 FIG S4 UV-vis spectral analysis of CcsBA WWD Ala variants. A 50-μg volume of the indicated GST affinity-purified CcsBA variant was used for UV-Vis spectra. Black, purified spectra; red, sodium dithionite reduction spectra. The level of pyridine hemochrome with 50 μg of protein (inset, green) with the percentage of copurified heme compared to the wild-type level is indicated. Data are representative of spectra from three independent purifications. Download FIG S4, JPG file, 1.2 MB. Copyright © 2018 Sutherland et al. 2018 Sutherland et al. This content is distributed under the terms of the Creative Commons Attribution 4.0 International license.

### Modeling the heme binding sites of CcsBA.

**FIG 6:**
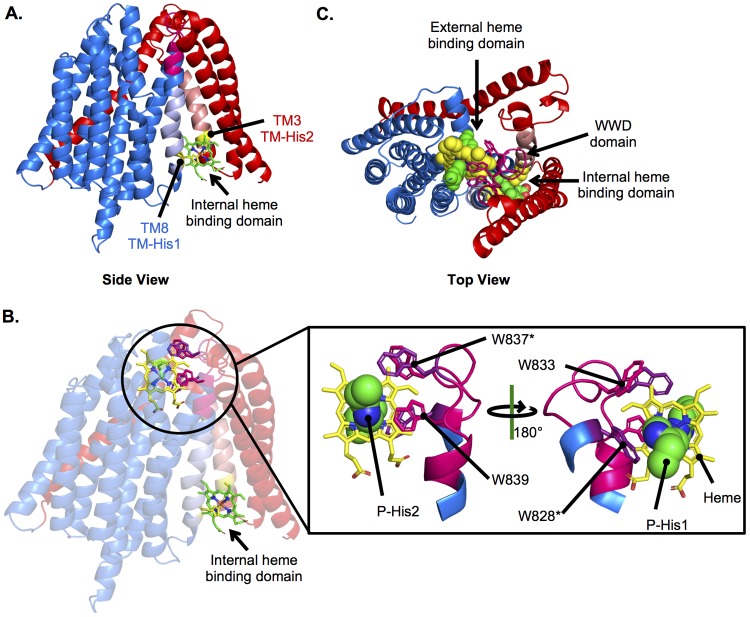
Modeling the CcsBA heme binding domains. The CcsBA model generated as described for Fig. 2 was used to model heme into the internal and external heme binding domains using experimentally determined constraints. CcsB is red, and CcsA is blue. (A) Heme (green) in the internal heme binding domain, liganded by TM-His1 and TM-His2 (yellow). (B) Heme in both the internal and external heme binding domains. The external heme binding domain is magnified to show heme (yellow) in the WWD domain (magenta) with conserved Trp labeled and liganded P-His1 and P-His2 (green). (C) Top view of heme in both the internal and external domains.

These results suggested that CcsBA contains two heme binding domains. To model CcsBA with heme, the CcsBA TM structural model (Fig. 2A) was further refined using the experimental constraints determined here. Heme in the TM domain was modeled with the constraint that TM-His1 and TM-His2 would function as axial ligands to the heme (Fig. 6A). The external heme binding domain (Fig. 6B) was modeled with the following constraints: (i) heme was positioned in the WWD domain with the vinyl groups exposed to the periplasm; (ii) W828C was near the 4-vinyl of heme (based on homology to CcmC W114C) (Fig. 4A) (30); (iii) W837C was near the 4-vinyl of heme (based on homology to CcmC W123C) (Fig. 4A) (30); (iv) P-His1 functioned as an axial ligand to heme; (v) P-His2 functioned as an axial ligand to heme on the opposite plane from P-His1; and (vi) the conserved Trp of the WWD domain directly interacted with heme (Fig. 6B). The resulting structure is very similar to the Rosetta-derived heme binding site of the CcmC WWD domain (30). Importantly, the working structure provides a framework for the mechanisms that underlie stereospecific attachment to the cyt *c* heme binding site, CXXCH (see Discussion). Although whether CcsBA can bind heme simultaneously in both the TM and external heme domains is unknown, a top view model suggested that heme in the TM domain can be transported and has access to the WWD/P-His binding domain (Fig. 6C). Thus, a path for heme transport in cyt *c* biogenesis begins to emerge.

### The large periplasmic region of CcsB is proteolyzed but retains function: evidence for a two-domain structure.

**FIG 7:**
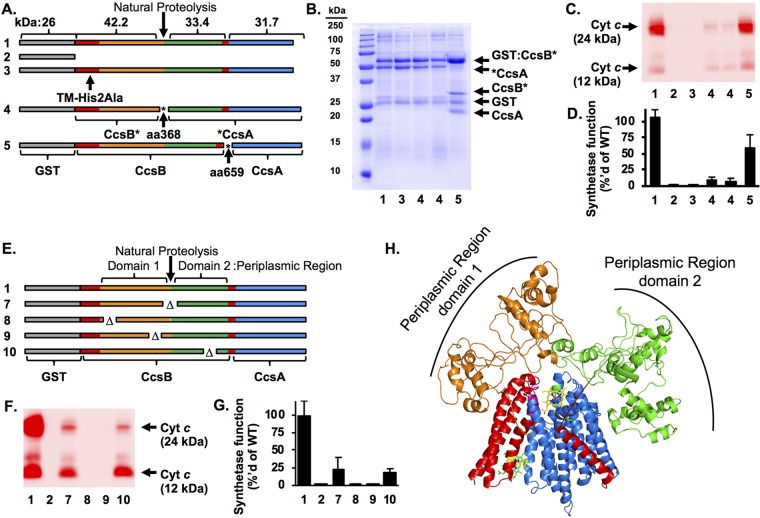
Analysis of the CcsBA periplasmic region. (A) Schematics of the GST:CcsBA artificial operon constructs (rows 1 to 5) are shown with polypeptide sizes indicated. The asterisks denote the insertion of an engineered stop/start cassette at the natural proteolysis site at residue 368 and the *ccsB*/*ccsA* junction at residue 659. GST tag, gray; CcsBA protein, white. Asterisks (*) indicate the insertion of an engineered stop/start cassette. Row 1, GST:CcsBA (WT); row 2, GST vector; row 3, GST:CcsBA TM-His2Ala; row 4, GST:CcsB*A aa368 (note that two independent clones were tested); row 5, GST:CcsB*A aa659. (B) GST affinity purifications of constructs 1 and 3 to 5. (C) CcsBA constructs were coexpressed with cyt *c*_4_; synthetase function was assessed by separation of cell lysate by SDS-PAGE, and cyt *c*_4_ levels were determined by heme staining. (D) Quantification of the data determined as described for panel C. WT data (sample 1) were normalized to 100%. Data represent results from three independent, triplicate experiments. (E) Schematics of GST:CcsBA deletions in the periplasmic region. Row 1, GST:CcsBA (WT) (cyt *c*_4_:His data are not diagrammed); row 7, GST:CcsBA (Δaa358–377); row 8, GST:CcsBA (Δaa100–109); row 9, GST:CcsBA (Δaa299-308); row 10, GST:CcsBA (Δaa536–545). (F) CcsBA constructs were coexpressed with cyt *c*_4_, and synthetase function was assessed as described for panel C. (G) Quantification of data from the experiment described in the panel F legend. WT data (sample 1) were normalized to 100%. Data represent results from three independent triplicate experiments. (H) Model of CcsBA (Fig. 2B) docked with model of periplasmic region. CcsB, red; CcsA, blue; periplasmic region domain 1, orange; periplasmic region domain 2, green.

Purified recombinant H. hepaticus CcsBA from E. coli is proteolyzed at residue 368 (Fig. 1A and 7A), resulting in two major polypeptides (GST:CcsB' and 'CcsA) (24) (Fig. 7B, lane 1). Similar proteolysis occurs in recombinant *CcsBA* from W. succinogenes (38). The fused *ccsBA* genes encode functional cyt *c* synthetases, but whether proteolysis inactivates this activity is unknown. Thus, we wanted to determine if eliminating proteolysis was possible. Attempts to mitigate proteolysis by expression of CcsBA in protease-deficient E. coli strains were unsuccessful (Fig. S5). Although most *ccsB* and *ccsA* genes in nature are not fused, attempts to functionally express these in E. coli have not been successful. For example, Bordetella pertussis *ccsA* was not expressed in E. coli (25), and Bacillus subtilis *resB* (*ccsB*) and *resC* (*ccsA*) were expressed but were not functional (35). Even though greater than 90% of recombinant fused CcsBA preparations are proteolyzed, it is feasible that the cyt *c* synthetase function is due to the small amount of full-length CcsBA.

10.1128/mBio.02134-18.6 FIG S5 CcsBA is proteolyzed. H. pylori GST:CcsBA was expressed in the following E. coli strains to determine if protein degradation could be mitigated: strains JM101 (lanes 5 and 6), BL21(DE3) (lanes 7 and 8 and lanes 17 and 18), TB1 (lanes 9 and 10), HB101 (lanes 11 and 12), and MG1655 (lanes 21 and 22). The following protease mutant strains were also tested: KS1000 (lanes 1 and 2; deficient in Prc protease) and ER2508 (lanes 3 and 4; deficient for the Lon protease); KS272 (lanes 13 and 14) is the parent strain in which a *degP* mutant was constructed (lanes 15 and 16). Membrane fractions of uninduced and induced strains were separated by SDS-PAGE and subjected to GST immunoblotting. Proteolyzed GST:CcsB′ fragments were identified in all strains. The size of full-length GST:CcsBA is indicated. Download FIG S5, JPG file, 1.0 MB. Copyright © 2018 Sutherland et al. 2018 Sutherland et al. This content is distributed under the terms of the Creative Commons Attribution 4.0 International license.

For future structure-function studies, we wanted to test if proteolysis affected cyt *c* synthetase function. Thus, artificial CcsBA operons were engineered to express separate CcsBA ORFs, initially split at the proteolysis junction (aa 368). We placed a translation stop/start cassette (stop codon/Shine-Dalgarno sequence/8-bp spacer/start codon) at the site of proteolysis (called “GST:CcsB*A aa368”) (Fig. 7A, row 4). Two independent clones were analyzed, and both analyses resulted in protein preparations mimicking the natural proteolysis site (Fig. 7B, lane 4). Note that the GST:CcsB* and *CcsA polypeptides were not stoichiometric (as in the wild type; Fig. 7B, lane 1), with *CcsA consistently at lower levels than GST:CcsB*; thus, some GST:CcsB was not complexed with CcsA, likely due to lower CcsA expression levels. However, enough of the CcsBA complex was present to test cyt *c* synthetase function. To determine if GST:CcsB*A aa368 is functional for cyt *c* maturation, the engineered genes were coexpressed with cyt *c*_4_ in E. coli Δ*ccm*. Levels of cyt *c* maturation were assessed by cell lysis and heme staining to detect covalently attached heme in cyt *c*_4_ (Fig. 7C). GST:CcsBA cyt *c* maturation levels were normalized to 100% (Fig. 7C and D, lane 1). A strain lacking CcsBA did not mature cyt *c* (Fig. 7C and D, lane 2). A CcsBA TM-His2Ala variant does not mature cyt *c* (24) (Fig. 7C and D, lane 3). Engineered GST:CcsB*A aa368 matured cyt *c* at ∼15% of wild-type level (Fig. 7C and D, lane 4). Because of the reduced levels of CcsBA complex in GST:CcsB*A aa368, we conclude there was at least 15% function and that proteolyzed H. hepaticus CcsBA retained function.

The stability and function of GST:CcsB*A aa368 illustrated the utility of the stop/start cassette approach for obtaining homogenous protein preparations of CcsBA and testing for domain composition. A similar approach had been used previously for DsbD, showing that DsbD can be split into three structural domains that retain function when expressed simultaneously (39). To further extend the analysis of CcsBA, a stop/start cassette was engineered at the junction of the *ccsB* and *ccsA* genes (GST:CcsB*A aa659) (Fig. 7A, row 5) to determine whether homogenous preparations of a nonfused CcsBA could be obtained, whether this preparation would be proteolyzed, and whether it would be functional with respect to mature cyt *c*. GST:CcsB*A aa659 was stable but was still proteolyzed at a location similar to that of wild-type fused CcsBA, yielding a product identical in size to GST:CcsB* (Fig. 7B, lane 5). GST:CcsB*A aa659 matures cyt *c* at approximately 60% of the wild-type level (Fig. 7C and D, lane 5). This represents the first case in which separate *ccsB* and *ccsA* genes have been functionally expressed in E. coli. Moreover, the results of the split-gene engineering suggested the CcsBA large periplasmic region is composed of two domains (see below). Each of the three polypeptides in GST:CcsB*A aa659 copurified over a glutathione column and appeared to form a stoichiometric complex.

**FIG 8:**
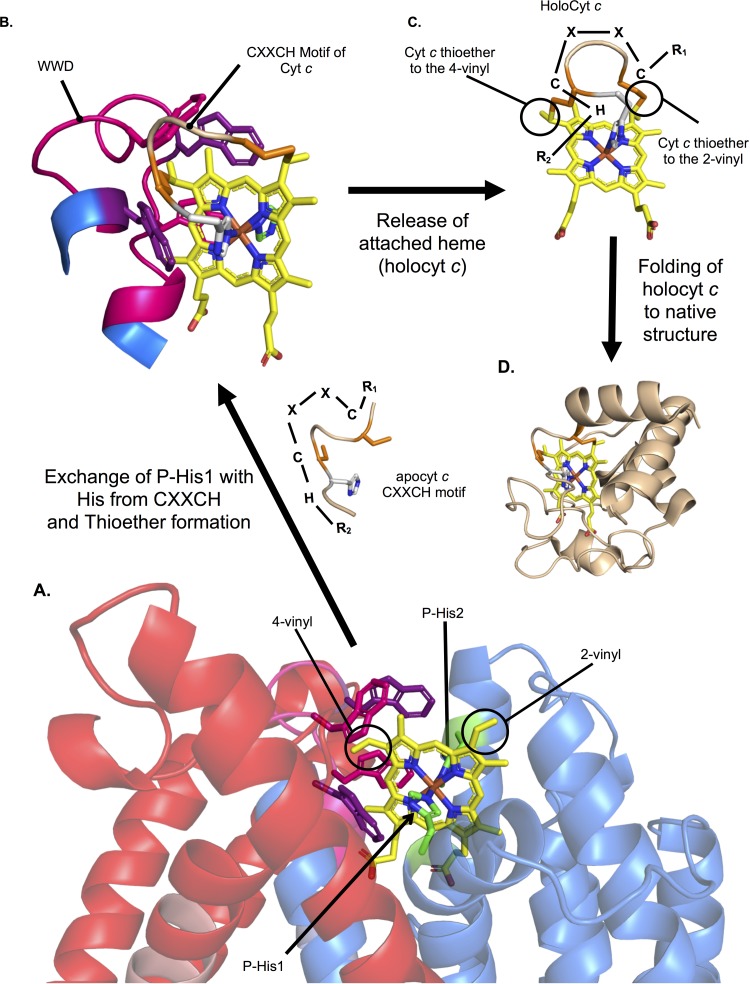
Model for heme attachment by CcsBA. (A) Heme is stereospecifically positioned in the WWD domain, liganded by P-His1 and P-His2 with the heme vinyl groups surface exposed to the periplasm. (B) The CXXCH motif of apocyt *c* is positioned near heme, allowing exchange of P-His1 for the new His ligand and thioether formation between the cysteine thiols and heme vinyl groups. (C) Heme, covalently attached to the CXXCH motif, is released from the WWD domain. (D) Cyt *c* folds around the heme. Note that the known human *c*ytochrome *c* (PDB 3ZCF, chain D) was used for illustrative purposes.

To further probe the role of the periplasmic region, a series of targeted deletions were engineered and assayed for stability, function, and role in heme binding. First, a 20-residue deletion spanning the natural proteolysis site was tested (CcsBA Δaa358–377) (Fig. 7E, row 7). This variant was still proteolyzed and retained ∼25% function (Fig. 7F and G, lane 7). This again suggested that the periplasmic region is composed of two domains with an accessible linker region that is prone to proteolysis. Residues in periplasmic domain 1 (CcsBA Δaa100–109 [Fig. 7E, row 8] and CcsBA Δaa229–308 [Fig. 7E, row 9]) and periplasmic domain 2 (CcsBA Δaa536–545 [Fig. 7E, row 10]) were analyzed. Periplasmic region 1 deletions were not functional (Fig. 7F and G, lanes 8 and 9), while the periplasmic domain 2 deletion retained ∼25% function (Fig. 7F and G, lane 10). All deletions were stable and bound heme at 50% to 100% of the wild-type level. Thus, we propose that the periplasmic region is not involved in heme transport and more likely functions in cyt *c* (CXXCH) presentation (see Discussion). Molecular modeling of the periplasmic regions (CcsBA aa 98 to 633) was attempted; however, convergence was not observed across the top 10 models. This was not unexpected, as the periplasmic region has little sequence conservation in CcsBA. However, all top models indicate a secondary structure that is >70% disordered. For illustrative purposes, the top-scoring periplasmic region model was docked with the CcsBA model (Fig. 7H). One feature obvious from the structure is that the periplasmic region could closely interact with cyt *c* substrates brought near heme in the WWD domain (see Fig. 7H and 8A).

## DISCUSSION

### Mechanisms of stereospecific heme attachment to cyt *c* (CXXCH) based on structure and function studies.

Heme transporters are poorly understood from the perspectives of heme trafficking across bilayer membranes and delivery of heme to acceptor proteins (40). Recent developments of heme sensors have contributed to understanding where intracellular heme resides (41–43), but knowledge of direct mechanisms for heme transport is still lacking. In these respects, integral membrane proteins required specifically for cyt *c* biogenesis (CcsBA and CcmC) are excellent systems for study. We have analyzed the predicted structure and propose mechanisms of heme export through the membrane to the external heme (WWD/P-His) domain by CcsBA. We show here that, unlike CcmC (and its acceptor CcmE), the acceptor (CXXCH) does not need to be present for heme export and binding to the external domain of CcsBA. We designate this domain the active site of the CcsBA synthetase, since the heme was unequivocally trapped here using the cysteine/heme crosslinking approach. The cysteine/heme crosslinking and P-His results were used to map and model heme in this domain (displayed in Fig. 8A). Using the known structure of heme attached to the CXXCH motif in cyt *c* (human cyt *c*), we propose a mechanism whereby exchange of P-His1 ligand with the histidine imidazole of CXXCH occurs at the active site (Fig. 8B). The result (Fig. 3B) whereby P-His1Gly can be corrected for function by free imidazole is consistent with this role. This ligand replacement would also help stereospecifically position the two thiols of CXXCH to form the two thioethers, the first cysteine corresponding to the 2-vinyl and the second cysteine corresponding to the 4-vinyl of heme (Fig. 8B and C) (see Fig. S3A in the supplemental material for chemistry). We have previously discussed how the functions of the two pairs of histidines (TM-His and P-His) include maintaining the heme iron in a reduced state (24, 44), a requirement for thioether attachment. Thus, the axial ligand exchange would occur without a change in reduction state.

After thioether attachment occurs, the holocyt *c* must be released from the CcsBA synthetase active site. We have demonstrated for the HCCS-mediated release of cyt *c* that formation of both thioether bonds is necessary for optimal release from the active site (10, 45). With CcsBA, a similar release mechanism may operate whereby distortion of the heme induced by the two thioether bonds is likely to further decrease the affinity of heme (now holocyt *c*) for the WWD domain. Thus, axial ligand exchange (cyt *c* His for the P-His) and thioether-induced distortion are proposed to play major roles in release. For most *c*-type cytochromes, folding to the native structure (Fig. 8D) occurs after heme attachment and release. The disordered periplasmic region (Fig. 7H) is well positioned in the CcsBA model both to aid binding of CXXCH at the active site (WWD domain) and to maintain the remainder of the apocyt *c* in an unfolded state until attachment occurs. The comprehensive set of CcsBA variants characterized here and structural models determined here will be useful for further testing many aspects of the mechanisms and structure.

## MATERIALS AND METHODS

### Bacterial growth conditions.

Escherichia coli strains were grown in Luria-Bertani broth (LB; Difco) at 24 C and 240 rpm with the following selective antibiotics and inducing reagents used at the indicated concentrations: carbenicillin, 50 μg/ml; chloramphenicol, 20 μg/ml; isopropyl β-d-1-thiogalactopyranoside (IPTG; Gold Biotechnology), 1.0 mM or 0.1 mM; arabinose (Alfa Aesar), 0.2% (wt/vol).

### Construction of strains and plasmids.

All cloning was performed using NEB-5α or XL-1 blue and a QuikChange II site-directed mutagenesis kit (Agilent Technologies) according to the manufacturer’s instructions. A complete list of strains, plasmids, and primers is provided in Table S2 in the supplemental material.

10.1128/mBio.02134-18.8 TABLE S2 List of strains, plasmids, and primers. Download Table S2, PDF file, 0.1 MB. Copyright © 2018 Sutherland et al. 2018 Sutherland et al. This content is distributed under the terms of the Creative Commons Attribution 4.0 International license.

### Protein purifications.

Affinity purifications of GST:CcsBA fusions were performed as previously described (24) with some modifications. E. coli strain C43 was used for protein expression (see Text S1 in the supplemental material for a complete description of the methods used).

### Heme staining and quantification.

Heme staining was performed as previously described (30, 46). Details are provided in Text S1.

### UV-visible absorption spectroscopy.

UV-visible absorption spectra were collected as described in reference 47 with the following modifications: 100 μg of protein in the buffer used for purification was used to collect spectra and to obtain quantitation of total heme levels using the Soret region. Pyridine hemochrome assays were performed as previously described (48) using 100 μg of protein in the buffer used for purification. Sodium dithionite powder was used to reduce protein for both UV-vis and pyridine spectra. Note that complete reduction of CcsBA was previously not accomplished (24); however, addition of excess dithionite allows complete reduction.

### Functional (heme attachment) assays.

The indicated CcsBA variants were coexpressed with *c*ytochrome *c*_4_:His (pRGK332) in strain C43 Δ*ccm*::KanR. Starter cultures were diluted 1:5 into 5 ml of LB with appropriate antibiotics and grown for 3 h at 37°C and 200 rpm. Cells were induced with 0.1 mM IPTG and 0.2% arabinose and grown for 3 h at 37°C and 200 rpm. Cells were collected by centrifugation and frozen at −80°C. Cells were lysed with 200 μl B-PER reagent (Thermo Fisher Scientific) per the manufacturer’s instructions, and 100-μg volumes of cells were separated by SDS-PAGE and analyzed for cyt *c* maturation by heme staining.

### Determination of heme redox potentials.

Redox potentials were determined by a modified version of the method previously described by Massey (49–51) as described in reference 47. The modifications are described in Text S1.

### Modeling of CcsBA.

CcsBA was modeled using GREMLIN and the Rosetta modeling suite (33, 34). See Text S1 for details.
