# Supplementary material for: Structure-Function Analysis of the Bifunctional CcsBA Heme Exporter and Cytochrome c Synthetase
Source: mBio. 2018 Dec 18;9(6):e02134-18. doi: 10.1128/mBio.02134-18 (PMC6299221; doi:10.1128/mBio.02134-18)
Supplement: TEXT S1 [file mbo006184227s1.pdf]

Supplemental Material

Title: Structure-function analysis of the bifunctional CcsBA heme exporter and cytochrome c synthetase

Authors: Molly C. Sutherland[a], Nate L. Tran[a], Dustin E. Tillman[a], Joshua M. Jarodsky[a,*], Jason Yuan[a] and Robert G. Kranz[a]#

[a]Department of Biology, Washington University in St. Louis, St. Louis, MO 63130, USA

[*]Present Address: Department of Chemistry, Portland State University, Portland, Oregon 97021, USA

#address correspondence to

Robert G. Kranz

Campus Box 1137

One Brookings Drive

St. Louis, MO 63130

Phone: +1 (314) 935-4278

Email: kranz@wustl.edu

Supplemental Material and Methods

Protein purifications

Affinity purifications of GST:CcsBA fusions were performed as previously described (1) with some modifications. *E. coli* strain C43 was used for protein expression. Starter cultures were diluted 1:200 in 1L LB with selective antibiotics, grown at 24C and 240 rpm to saturation (~16-18 hours), induced with 1 mM IPTG for 6 hours, harvested by centrifugation and cell pellets were stored at -80C. Cells were resuspended in Resin buffer (20 mM Tris pH8, 100 mM NaCl), supplemented with 1 mM phenylmethansulfonyl fluoride (PMSF, Sigma-Aldrich) and 1 mg/mL egg white lysozyme (Sigma-Aldrich). Cells were lysed (Branson250 sonicator), cleared of cell debris by centrifugation at 24000g for 30 minutes, 4C. Soluble and membrane fractions were separated by high-speed ultracentrifugation for 45 minutes at 100000g, 4C. Membrane pellets were solubilized in Resin buffer with 1% *n*-dodecyl-β-d-maltopyranoside (DDM; Anatrace). Proteins were affinity purified by batch method with glutathione agarose (Pierce), washed by gravity flow, eluted in 4 mL Resin buffer with 0.02% DDM and 20 mM L-glutathione (Sigma-Aldrich) and concentrated in a 100 kDa Amicon filter. Proteins were separated by SDS-PAGE and visualized by total protein stain with Coomassie Blue.

Heme stains and quantification

Heme stains were performed as previously described (2, 3). Briefly, proteins were transferred to 0.45 µm Amersham Protran nitrocellulose (GE Healthcare Life Sciences) or 0.2 µm Immobilon P^{SQ} PVDF (Millipore) and imaged on a LAS-1000 Plus (Fujifilm-GE Healthcare) or LI-CORE odyssey Fc (LI-COR Biosciences). Heme abundance was

quantified with ImageJ (4) or Image Studio Lite Version 5.2 software (LI-COR Biosciences).

Determination of heme redox potentials

Redox potentials were determined by a modified Massey method (5–7) as described in (8) with the following modifications: Samples were buffer exchanged to remove glutathione. Redox titrations were performed in 20 mM Tris pH8, 100 mM NaCl, 0.02% DDM and pH typically increased to pH8.3 during the reaction. The absorbance change of the heme Soret was monitored at 426 nm and reduction of the reference dye Nile Blue at 630 nm.

CcsBA Modeling

Sequence Construction and GREMLIN Analysis

The sequence of naturally fused *H. hepaticus* CcsBA was used for modeling. Jackhmmer analysis resulted in a low number of homologous sequences per length, or Seq/Len, throughout the large CcsB periplasmic region (aa 98-633), thus 36 continuous residues with the greatest sequence conservation were chosen to link TM3 and TM4. As described by Kamisetty *et al.*, Seq/Len values of 5.0 and above directly correlate to optimal GREMLIN performance, generating more accurate coevolution-based distance constraints which help improve the accuracy of modeled structures (9). As a starting point, replacing the large CcsB periplasmic region with the 36 residue periplasmic region linker increased the overall Seq/Len value from 0.375 to 1.432 using the GREMLIN “monomer protocol” (9). The final sequence used for this modeling is

comprised of residues 1-97 (TMs 1-3), 286-321 (36 residue periplasmic region linker), 634-935 (TMs 4-10) of the *H. hepaticus* fused CcsBA sequence with no gaps in the final sequence. Note that CcsBA exists in nature as a fused orf (as in *H. hepaticus*), or more often as two separate genes, *ccsB* and *ccsA* (10). Because the GREMLIN monomer protocol appeared to exclude some separate CcsB and CcsA sequences from coevolution analysis due to its coverage filter, the query sequence was split into CcsB (residues 1-97, 286-321, 634-658) and CcsA (residues 658-935) to be input to the GREMLIN "complex protocol" (9, 11). Using the complex protocol further improved the overall Seq/Len value from 1.432 to 4.84. Because of the low number of homologous sequences per length for the 36 residue periplasmic linker, co-evolved residues and their constraints from this linker were not used during modeling.

Fragment Generation

The Robetta webserver was used to generate 3 and 9 amino acid structural fragments and the PSIPRED secondary structure prediction (12).

Trans-Membrane Domain Prediction

The OCTOPUS webserver was used to generate a trans-membrane region prediction specifying which residues would be located within the membrane (13) .The OCTOPUS predictions similarly matched both the PSIPRED predictions and the experimental topology determined by prior experiments (1, 14, 15) with the exception of the hydrophobic patches.

Model Building

For the initial global sampling, the Rosetta *ab initio* procedure in combination with GREMLIN constraints was used as described in (16) for trans-membrane proteins. An additional bounded constraint was used to restrain the distance between the beta carbons of the two TM-His residues (H83 and H858) to a range of 10 to 12 angstroms, typical for bis-histidine heme proteins. These constraints were used during both the coarse-grained sampling and full-atom refinement stages. 20,000 *ab initio* models were generated. The top ten scoring models by sum of the Rosetta score and the constraint energy were compared for convergence. These models converged over substructures, namely TMs 5, 6, 7, 8, 9, and the hydrophobic patches (overall TM-score > 0.5) (17, 18). Following a general procedure as outlined in (16) the top five models were recombined to a pool of 1,000 structures using the Rosetta hybridization protocol (19). The top ten models were scanned for placement of local structures in the correct relative topology determined by the OCTOPUS and PSIPRED predictions and the previously mentioned experimentally determined topology. Models which fulfilled this requirement were used as the template structures for the next round of hybridization. Iterative refinement by hybridization (20) guided by topology was performed until all top ten scoring models converged (TM-score > 0.7) and accurately reflected correct topology. The top scoring model was used for further modeling with heme.

Modeling with Heme

A procedure previously used to model the CcmC periplasmic heme-binding site was adapted to model the homologous periplasmic heme-binding site of CcsBA (3). The

following loops were removed to prevent bias from the starting structure: residues 738-762, 828-843, and 892-907. These loops respectively correspond to: P-His loop 1 (H761), WWD domain, and P-His loop 2 (H897). HMY (heme-like molecule), with the vinyl and propionates replaced by methyl groups, was generated. The Rosetta hybridization protocol (19) was used to model the removed loops around HMY. During coarse-grained sampling, a bounded constraint between 10 to 12 angstroms was used to restrain the distance between the beta carbons of the two P-His residues. During full-atom refinement, harmonic constraints were used. These include distance constraints between the NE2 atoms of the P-His and FE atom of HMY (mean: 2 angstroms, stdev 0.1), angle constraints between N1-FE-NE2, N2-FE-NE2, N3-FE-NE2, and N4-FE-NE2 (mean: 90 degrees, stdev: 10), and a dihedral constraint for each P-His between CG-CD2-NE2-FE (mean: 180 degrees, stdev: 10). 2,000 models were generated. Heme placement converged in the top ten scoring models (TM-score > 0.7). These models were then screened for correct orientation of the HMY: propionates facing down, 2-vinyl group exposed, and 4-vinyl interacting with W828 and W837.

The top scoring model that satisfied this condition was selected for further modeling with the complete heme. Residues 828-843 were removed from the structure to prevent bias from the starting model. Ambiguous sigmoidal constraints (mean: 5 angstroms, slope: 4) between all side-chain carbons of W828, W833, W837, and W839 and the aromatic carbons of heme were used during both coarse-grained and full-atom stages of the Rosetta hybridization protocol. This was done to bias orientation of the tryptophan residues to maximize aromatic ring stacking between the residues and heme. In

addition to the sigmoidal, harmonic, and bounded constraints described before, additional bounded constraints were added between K842 and propionate carbons (0-4 angstroms), as well as between W828, W837 and the 4-vinyl carbons (0-4 angstroms). Further bounded constraints were added between W833 and N4 pyrrole and between W839 and N3 pyrrole (0-6 angstroms). 1,500 models were generated. The top ten models converged (TM-score > 0.7) and the top scoring model was selected for further modeling of heme into the TM heme-binding site.

An additional full heme was loaded into this top scoring model and harmonic constraints that were used for P-His modeling (H761 and H897) were added between the TM-His residues (H83 and H858) and this new heme molecule. The TM-His bounded constraint as described above was retained to restrain the residues together during coarse-grained modeling and full-atom refinement while the harmonic constraints were used only during full-atom refinement. 1,500 models were generated using Rosetta hybridization (19). The top ten models converged (TM-score > 0.7) and the top scoring model was selected to be shown in this paper.

Modeling the Large Periplasmic Region

For illustrative purposes only, the large CcsB periplasmic region (aa 98-633) was modeled independently from the remainder of CcsBA. The sequence corresponding to this region (aa 98-633) was input into both the GREMLIN and Robetta webservers to generate inter-residue distance constraints, structural fragments, and the PSIPRED secondary structure predictions. The Rosetta *ab initio* procedure in combination with

GREMLIN constraints was used as described in (16). 20,000 models were generated. The top ten models did not converge (TM-score < 0.3). The top scoring model was selected to replace the previously mentioned 36 residue periplasmic linker in the CcsBA structure. Because of the low number of homologous sequences per length as determined by Jackhmmer analysis and the large size of the region (> 500aa), the models are not expected to be accurate at the level of converged structures.

Supplemental References

1. Frawley ER, Kranz RG. 2009. CcsBA is a cytochrome *c* synthetase that also functions in heme transport. Proc Natl Acad Sci 106:10201–10206.

2. Feissner R, Xiang Y, Kranz RG. 2003. Chemiluminescent-based methods to detect subpicomole levels of *c*-type cytochromes. Anal Biochem 315:90–94.

3. Sutherland MC, Jarodsky JM, Ovchinnikov S, Baker D, Kranz RG. 2018. Structurally Mapping Endogenous Heme in the CcmCDE Membrane Complex for Cytochrome *c* Biogenesis. J Mol Biol 430:1065–1080.

4. Rasband WS. 1997. ImageJ. US Natl Inst Health Bethesda MD Available at:http://imagej.nih.gov/ij/.

5. Massey V. 1991., p. 59–66. *In* Flavins and Flavoproteins. Walter de Gruyter & Co., New York.

6. Efimov I, Papadopoulou ND, McLean KJ, Badyal SK, Macdonald IK, Munro AW, Moody PCE, Raven EL. 2007. The redox properties of ascorbate peroxidase. Biochemistry 46:8017–8023.

7. Efimov I, Parkin G, Millett ES, Glenday J, Chan CK, Weedon H, Randhawa H, Basran J, Raven EL. 2014. A simple method for the determination of reduction potentials in heme proteins. FEBS Lett 588:701–704.

8. Sutherland MC, Rankin JA, Kranz RG. 2016. Heme Trafficking and Modifications during System I Cytochrome *c* Biogenesis: Insights from Heme Redox Potentials of Ccm Proteins. Biochemistry 55:3150–3156.

9. Kamisetty H, Ovchinnikov S, Baker D. 2013. Assessing the utility of coevolution-based residue-residue contact predictions in a sequence- and structure-rich era. Proc Natl Acad Sci 110:15674–15679.

10. Kranz RG, Richard-Fogal C, Taylor J-S, Frawley ER. 2009. Cytochrome *c* biogenesis: mechanisms for covalent modifications and trafficking of heme and for heme-iron redox control. Microbiol Mol Biol Rev 73:510–528.

11. Ovchinnikov S, Kamisetty H, Baker D. 2014. Robust and accurate prediction of residue–residue interactions across protein interfaces using evolutionary information. eLife 3:e02030.

12. Jones DT. 1999. Protein secondary structure prediction based on position-specific scoring matrices. J Mol Biol 292:195–202.

13. Viklund H, Elofsson A. 2008. OCTOPUS: improving topology prediction by two-track ANN-based preference scores and an extended topological grammar. Bioinformatics 24:1662–1668.

14. Goldman BS, Beck DL, Monika EM, Kranz RG. 1998. Transmembrane heme delivery systems. Proc Natl Acad Sci 95:5003–5008.

15. Dreyfuss BW, Hamel PP, Nakamoto SS, Merchant S. 2003. Functional Analysis of a Divergent System II Protein, Ccs1, Involved in *c* -Type Cytochrome Biogenesis. J Biol Chem 278:2604–2613.

16. Ovchinnikov S, Kinch L, Park H, Liao Y, Pei J, Kim DE, Kamisetty H, Grishin NV, Baker D. 2015. Large-scale determination of previously unsolved protein structures using evolutionary information. eLife 4:e09248.

17. Zhang Y, Skolnick J. 2004. Scoring function for automated assessment of protein structure template quality. Proteins Struct Funct Bioinforma 57:702–710.

18. Xu J, Zhang Y. 2010. How significant is a protein structure similarity with TM-score = 0.5? Bioinformatics 26:889–895.

19. Song Y, DiMaio F, Wang RY-R, Kim D, Miles C, Brunette T, Thompson J, Baker D. 2013. High-Resolution Comparative Modeling with RosettaCM. Structure 21:1735–1742.

20. Park H, Ovchinnikov S, Kim DE, DiMaio F, Baker D. 2018. Protein homology model refinement by large-scale energy optimization. Proc Natl Acad Sci 115:3054–3059.
