# Supplementary figures and images for: Structure-Function Analysis of the Bifunctional CcsBA Heme Exporter and Cytochrome c Synthetase
Source: mBio. 2018 Dec 18;9(6):e02134-18. doi: 10.1128/mBio.02134-18 (PMC6299221; doi:10.1128/mBio.02134-18)

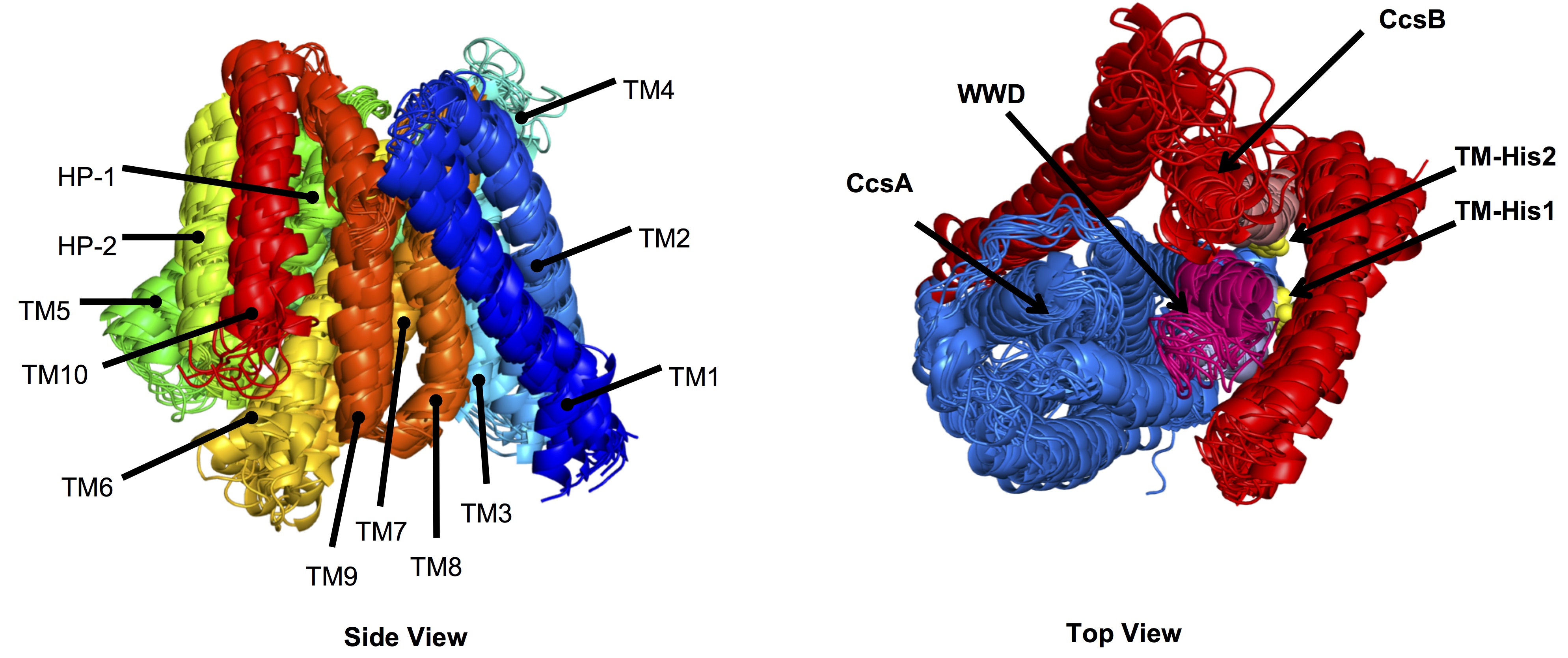

Supplement: FIG S1 [file mbo006184227sf1.jpg]

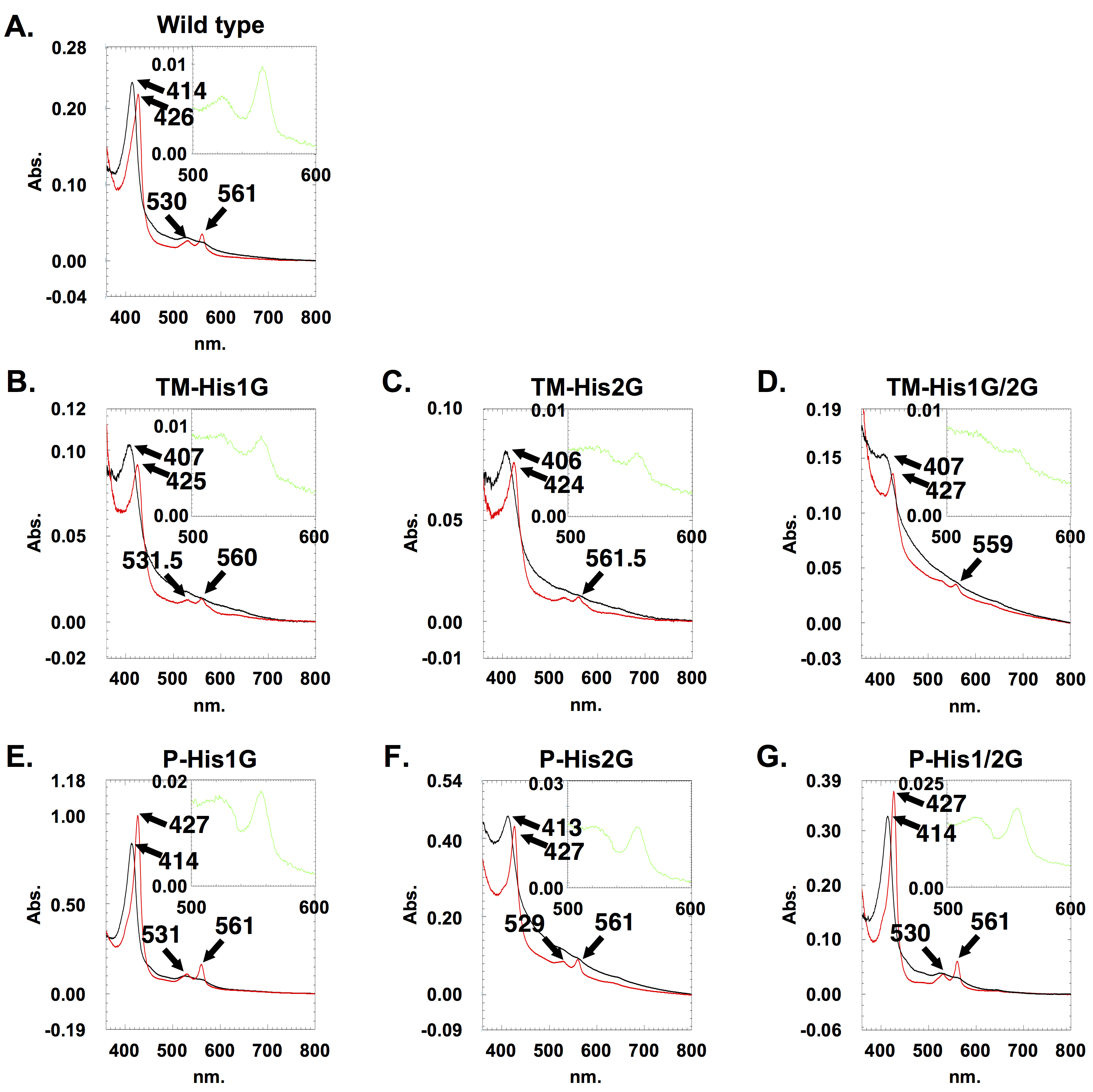

Supplement: FIG S2 [file mbo006184227sf2.jpg]

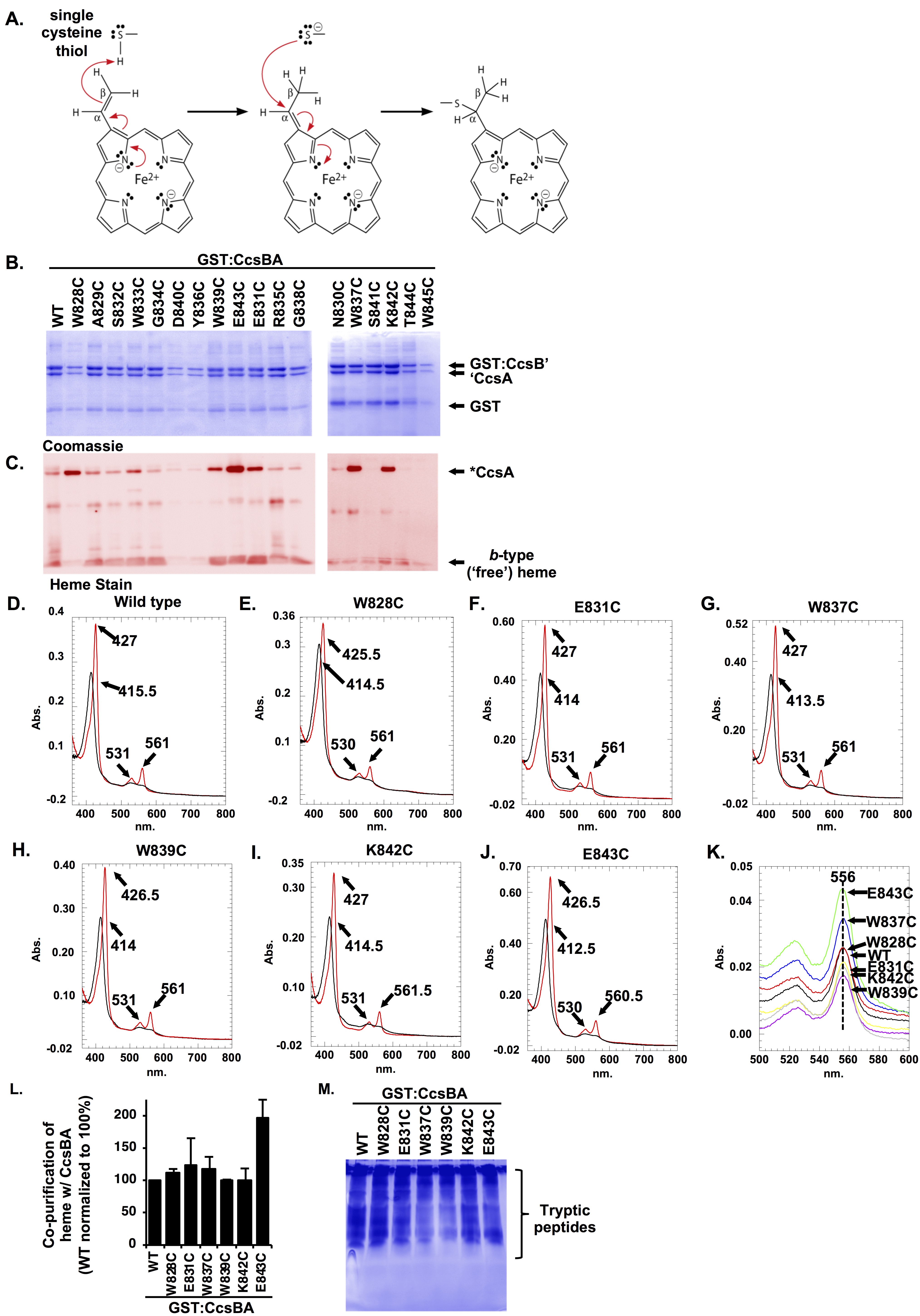

Supplement: FIG S3 [file mbo006184227sf3.jpg]

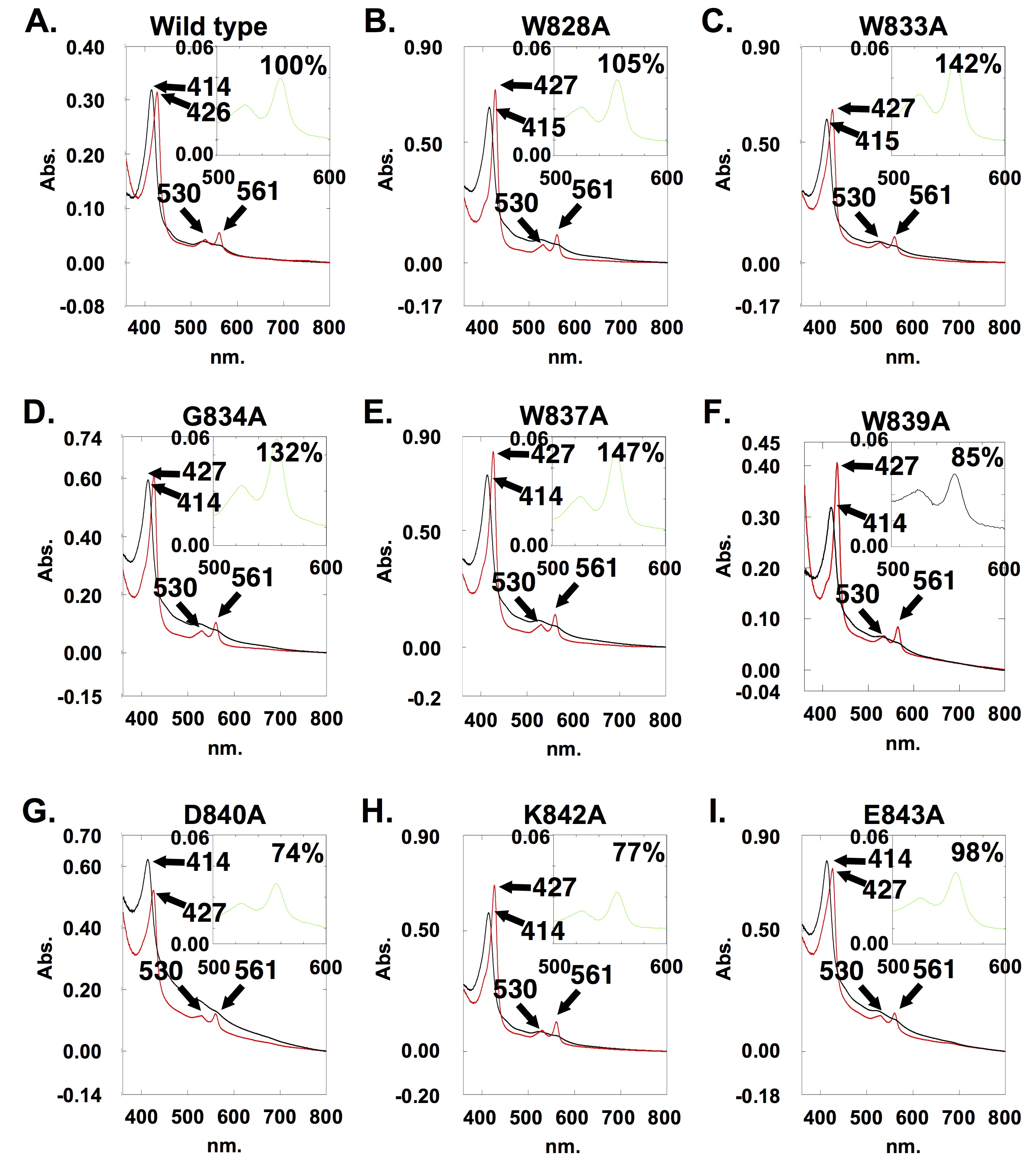

Supplement: FIG S4 [file mbo006184227sf4.jpg]

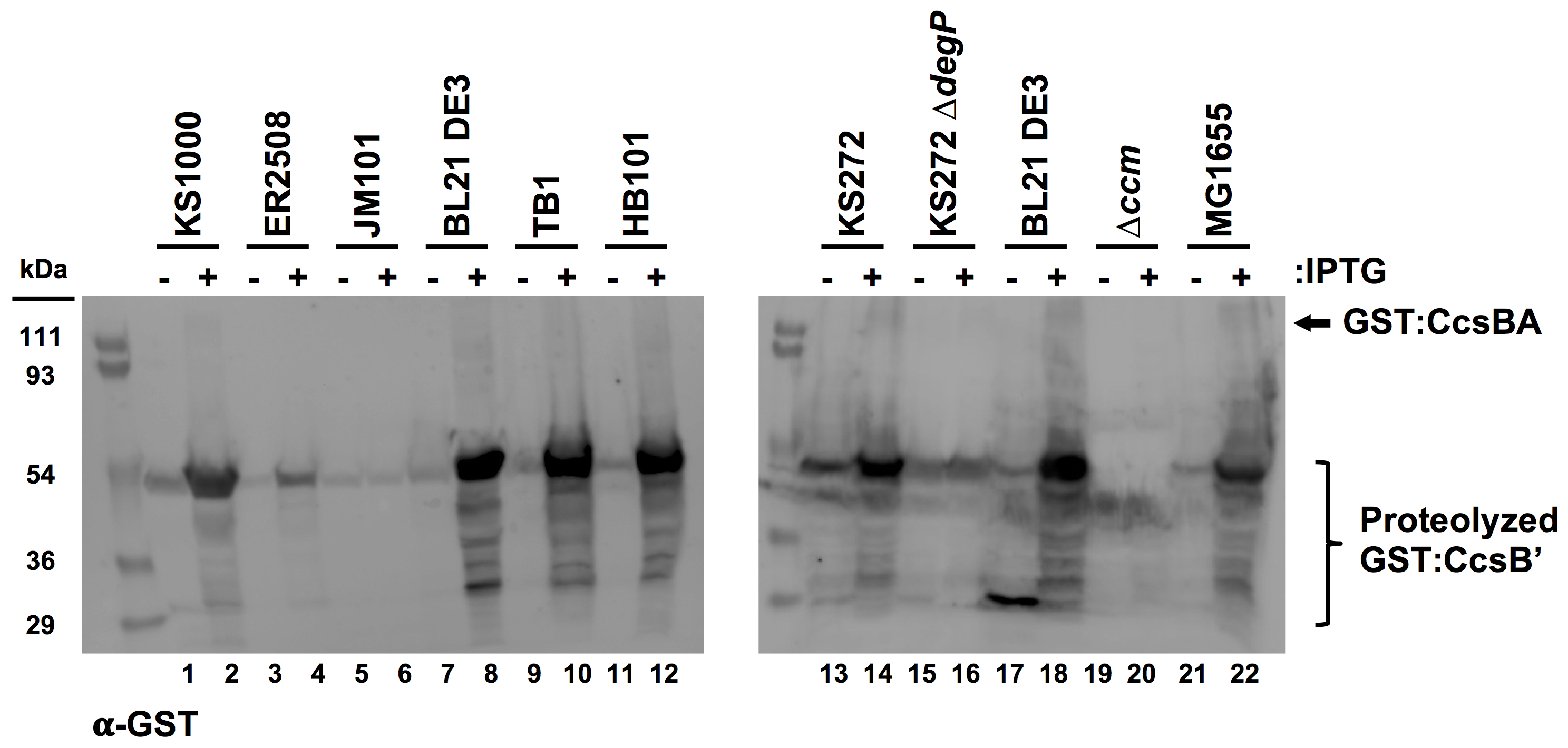

Supplement: FIG S5 [file mbo006184227sf5.jpg]
