# Supplementary material for: Structure-Function Analysis of the Bifunctional CcsBA Heme Exporter and Cytochrome c Synthetase
Source: mBio. 2018 Dec 18;9(6):e02134-18. doi: 10.1128/mBio.02134-18 (PMC6299221; doi:10.1128/mBio.02134-18)
Supplement: TABLE S1 [file mbo006184227st1.pdf]

Table S1. CcsBA Heme Redox Potentials

Description	Redox Potential (Mv)
GST:CcsBA	-114.5 +/- 0.6
GST:CcsBA (P-His1G)	-119.0 +- 6.0
GST:CcsBA (P-His2G)	-122.41 +/- 5.7
GST:CcsBA (W833A)	-125.5 +/- 10.6
