# Supplementary material for: Structure-Function Analysis of the Bifunctional CcsBA Heme Exporter and Cytochrome c Synthetase
Source: mBio. 2018 Dec 18;9(6):e02134-18. doi: 10.1128/mBio.02134-18 (PMC6299221; doi:10.1128/mBio.02134-18)
Supplement: TABLE S2 [file mbo006184227st2.pdf]

Table S2. Relevant Strains, Plasmids and Primers Employed in this Study

Strain, Plasmid	Description	Reference
E. coli		
NEB 5-α	fhuA2 Δ(argF-lacZ)U169 phoA glnV44 Φ80 Δ(lacZ)M15 gyrA96 recA1 relA1 endA1 thi-1 hsdR17	
C43(DE3)	F – ompT hsdSB (rB- mB-) gal dcm (DE3)	*Miroux and Walker, 1996*
RK103	*E. coli* MG1655 Δ*ccm::kan*^{R}, deleted for all *ccm* genes	*Feissner et al, 2006*
MS36	C43 Δ*ccm::kan*^{R}, deleted for all ccm genes	This study
Plasmid		
pRGK332	pBAD *Bordetella pertussis* cytochrome c4:His	*Feissner et al, 2006*
pRGK368	pGEX *Helicobacter hepaticus* GST:CcsBA	*Richard-Fogel et al, 2007*
pMCS5	pGEX GST:CcsBA (H858A aka TM-His1A)	*Frawley and Kranz, 2009*
pMCS10	pGEX GST:CcsBA (H83G aka TM-His2G)	This Study
pMCS39	pGEX GST:CcsBA (H761G aka P-His1G)	This Study
pMCS81	pGEX GST:CcsBA (H83A aka TM-His2A)	*Frawley and Kranz, 2009*
pMCS84	pGEX GST:CcsBA (H858G aka TM-His1G)	This Study
pMCS106	pGEX GST:CcsBA (H761A aka P-His1G)	*Frawley and Kranz, 2009*
pMCS107	pGEX GST:CcsBA (H897A aka P-His2A)	*Frawley and Kranz, 2009*
pMCS109	pGEXT GST:CcsBA (H83G/H858G aka TM-His2G/TM-His1G)	This Study
pMCS147	pGEX GST:CcsBA (H897G aka P-His2G)	This Study
pMCS154	pGEX GST:CcsBA (H761G/H897G aka P-His1G/P-His2G)	This Study
pMCS194	pGEX GST:CcsBA (W833A)	This Study
pMCS200	pGEX GST:CcsBA (G834A)	This Study
pMCS201	pGEX GST:CcsBA (W837A)	This Study
pMCS202	pGEX GST:CcsBA (W839A)	This Study
pMCS203	pGEX GST:CcsBA (D840A)	This Study
pMCS204	pGEX GST:CcsBA (E843A)	This Study
pMCS217	pGEX GST:CcsBA (K842A)	This Study
pMCS254	pGEX GST:CcsBA (W828A)	This Study
pMCS270	pGEX GST:CcsB*A (aa 659)	This Study
pMCS272	pGEX GST:CcsB*A (aa 356) clone 1	This Study
pMCS275	pGEX GST:CcsB*A (aa 356) clone 2	This Study
pMCS289	pGEX GST:CcsBA(W828C)	This Study
pMCS290	pGEX GST:CcsBA(A829C)	This Study
pMCS291	pGEX GST:CcsBA(S832C)	This Study
pMCS292	pGEX GST:CcsBA(W833C)	This Study
pMCS293	pGEX GST:CcsBA(G834C)	This Study
pMCS294	pGEX GST:CcsBA(D840C)	This Study
pMCS295	pGEX GST:CcsBA(Y836C)	This Study
pMCS296	pGEX GST:CcsBA(W839C)	This Study
pMCS297	pGEX GST:CcsBA(E843C)	This Study
pMCS298	pGEX GST:CcsBA(E831C)	This Study

pMCS299	pGEX GST:CcsBA(R835C)	This Study	
pMCS300	pGEX GST:CcsBA(G838C)	This Study	
pMCS313	pGEX GST:CcsBA(K842C)	This Study	
pMCS316	pGEX GST:CcsBA(W837C)	This Study	
pMCS317	pGEX GST:CcsBA(N830C)	This Study	
pMCS318	pGEX GST:CcsBA(S841C)	This Study	
pMCS319	pGEX GST:CcsBA(T844C)	This Study	
pMCS320	pGEX GST:CcsBA(W845C)	This Study	
pMCS585	pGEX GST:CcsBA (Δaa358-377)	This Study	
pMCS586	pGEX GST:CcsBA (Δaa100-109)	This Study	
pMCS587	pGEX GST:CcsBA (Δaa299-308)	This Study	
pMCS588	pGEX GST:CcsBA (Δaa536-545)	This Study	

Oligonucleotide	Sequence (5' --> 3')	Purpose	Template
CcsBA 1104ins - F	atagcttatgcctcaacacaatgaaggaggacttcaccatgtcacaaactgacttccagc	pMCS272 cloning	pRGK368
		pMCS275 cloning	pRGK368
CcsBA 1104ins - R	gctggaagtcagtttgtgacatggtgaagtcctccttcattgtgttgaggcataagctat	pMCS272 cloning	pRGK368
		pMCS275 cloning	pRGK368
CcsBA H83G - F	gataaaagaggcgcccaagagcaa	pMCS10 cloning	pRGK368
CcsBA H83G - R	ctattttctccgcgggttctcgtt	pMCS10 cloning	pRGK368
CcsBA H858G - F	gcaattatcttaggcttgcgctttgttgtgcc	pMCS84 cloning	pRGK368
		pMCS109 cloning	pMCS10
CcsBA H858G - R	cgttaatagaatccgaacgcgaaacaacacgg	pMCS84 cloning	pRGK368
		pMCS109 cloning	pMCS10
MSP5	gtgcttaaatcttattggctcaacattggcgtctccgtcatca	pMCS39 cloning	pRGK368
		pMCS154 cloning	pMCS147
MSP6	tgatgacggagacgccaatgttgagccaataagatttaagcac	pMCS39 cloning	pRGK368
		pMCS154 cloning	pMCS147
MSP7	ttattatctcacaggtatgggcagctatgccgcaggagaa	pMCS147 cloning	pRGK368
MSP8	ttctcctgcggcatagctgcccatacctgtgagataataa	pMCS147 cloning	pRGK368
MSP37	gccgcctcttttatcgttatcatcattggtgcggg	pMCS81 cloning	pRGK368
MSP38	cccgcaccaatgatgataacgataaaagaggcggc	pMCS81 cloning	pRGK368
MSP69	ccaatatctgcccgcagattcattagcccaaatgccacca	pMCS194 cloning	pRGK368
MSP70	tggtggcatttgggctaatgaatctgcgggcagatattgg	pMCS194 cloning	pRGK368
MSP71	ccagccccaatatctggcccaagattcattagc	pMCS200 cloning	pRGK368
MSP72	gctaatgaatcttgggccagatattggggctgg	pMCS200 cloning	pRGK368
MSP73	tagaatcccagcccgcatatctgccccaagattcattagcc	pMCS201 cloning	pRGK368
MSP74	ggctaatgaatcttggggcagatatgcgggctgggattcta	pMCS201 cloning	pRGK368
MSP75	ccaagtttctttagaatccgcgccccaatatctgccccaa	pMCS202 cloning	pRGK368
MSP76	ttggggcagatattggggcgcggattctaaagaaacttgg	pMCS202 cloning	pRGK368
MSP77	cccaagtttctttagaagcccagccccaatatctg	pMCS203 cloning	pRGK368
MSP78	cagatattggggctgggcttctaaagaaacttggg	pMCS203 cloning	pRGK368
MSP79	aagtgcccaagtttctgcagaatcccagccccaatatctgc	pMCS217 cloning	pRGK368
MSP80	gcagatattggggctgggattctgcagaaacttgggcactt	pMCS217 cloning	pRGK368

MSP81	ctaataagtgcccaagttgctttagaatcccagcccc	pMCS204 cloning	pRGK368
MSP82	ggggctgggattctaaagcaacttgggcacttattag	pMCS204 cloning	pRGK368
MSP83	gccccaatatctgccccaagattcattagccgcaatgccaccaaggaagttccctacacttagc	pMCS254 cloning	pRGK368
MSP84	gctaagtgtagggaacttccttggtggcattgcggctaatgaatcttggggcagatattggggc	pMCS254 cloning	pRGK368
MSP85	tgataggaattccaaaaatactcctacatgaaggaggacttcaccatgctccgcttattcac	pMCS270 cloning	pRGK368
MSP86	gtgaataagcggacgcatggtgaagtcctccttcatgtaggagtatttttgagaattcctacaa	pMCS270 cloning	pRGK368
MSP87	ttccttggtggcatttgcgctaatgaatcttgggg	pMCS289 cloning	pRGK368
MSP88	ccccaagattcattagcgcaaatgccaccaaggaa	pMCS289 cloning	pRGK368
MSP89	cttccttggtggcatttggtgtaatgaatcttggggcaga	pMCS290 cloning	pRGK368
MSP90	tctgccccaagattcattacaccaaatgccaccaaggaag	pMCS290 cloning	pRGK368
MSP91	ccttggtggcatttgggcttgtgaatcttggggcag	pMCS317 cloning	pRGK368
MSP92	ctgccccaagattcacaagcccaaatgccaccaagg	pMCS317 cloning	pRGK368
MSP93	ttccttggtggcatttgggctaattgctcttggggcagatattgg	pMCS298 cloning	pRGK368
MSP94	ccaatatctgccccaagagcaattagcccaaatgccaccaaggaa	pMCS298 cloning	pRGK368
MSP95	ggtggcatttgggctaatgaatgttggggcagata	pMCS291 cloning	pRGK368
MSP96	tatctgccccaacattcattagcccaaatgccacc	pMCS291 cloning	pRGK368
MSP97	tgggctaatgaatcttgcggcagatattggggc	pMCS292 cloning	pRGK368
MSP98	gccccaatatctgccgcaagattcattagccca	pMCS292 cloning	pRGK368
MSP99	gggctaatgaatcttggtgcagatattggggctgg	pMCS293 cloning	pRGK368
MSP100	ccagccccaatatctgcaccaagattcattagccc	pMCS293 cloning	pRGK368
MSP101	gctaatgaatcttggggctgctattggggctgggattct	pMCS299 cloning	pRGK368
MSP102	agaatcccagccccaatagcagccccaagattcattagc	pMCS299 cloning	pRGK368
MSP103	gctaatgaatcttggggcagatgttggggctggga	pMCS295 cloning	pRGK368
MSP104	tcccagccccaacatctgccccaagattcattagc	pMCS295 cloning	pRGK368
MSP105	atcttggggcagatattgcggctgggattctaaa	pMCS316 cloning	pRGK368
MSP106	tttagaatcccagccgcaatatctgccccaagat	pMCS316 cloning	pRGK368
MSP107	cttggggcagatattggtgctgggattctaaaga	pMCS300 cloning	pRGK368
MSP108	tctttagaatcccagcaccaatatctgccccaag	pMCS300 cloning	pRGK368
MSP109	ggcagatattggggctgcgattctaaagaaacttg	pMCS296 cloning	pRGK368
MSP110	caagtttctttagaatcgcagccccaatatctgcc	pMCS296 cloning	pRGK368
MSP111	gggcagatattggggctggtgttctaaagaaacttgggca	pMCS294 cloning	pRGK368
MSP112	tgcccaagtttctttagaacaccagccccaatatctgccc	pMCS294 cloning	pRGK368
MSP113	atattggggctgggattgtaaagaaacttgggcac	pMCS318 cloning	pRGK368
MSP114	gtgcccaagtttctttacaatcccagccccaatat	pMCS318 cloning	pRGK368
MSP115	gcagatattggggctgggattcttgcgaaacttgggcacttattagtat	pMCS313 cloning	pRGK368
MSP116	atactaataagtgcccaagtttcgcaagaatcccagccccaatatctgc	pMCS313 cloning	pRGK368
MSP117	ggcagatattggggctgggattctaaatgcacttgggcacttattagt	pMCS297 cloning	pRGK368
MSP118	actaataagtgcccaagtgcatttagaatcccagccccaatatctgcc	pMCS297 cloning	pRGK368
MSP119	agatattggggctgggattctaaagaatgttgggcacttattag	pMCS319 cloning	pRGK368
MSP120	ctaataagtgcccaacattctttagaatcccagccccaatatct	pMCS319 cloning	pRGK368
MSP121	ctgggattctaaagaaacttgcgcacttattagtattggtgta	pMCS320 cloning	pRGK368
MSP122	tacaccaatactaataagtgcgcaagtttctttagaatcccag	pMCS320 cloning	pRGK368

MSP273	cttgtaaaggtggtatttcattttccaaagcataacaaagtgcactacaaagtgc	pMCS585 cloning	pRGK368
MSP274	gcactttgtagtgcactttgttatgctttggaaaatgaaataccacctttacaag	pMCS585 cloning	pRGK368
MSP275	tggtgcgggtattactcgttattatggacaaagcgtga	pMCS586 cloning	pRGK368
MSP276	tcacgctttgtccataataacgagtaatacccgcacca	pMCS586 cloning	pRGK368
MSP277	ccctgatgaaaaaggctctattcctacatacataggctatgc	pMCS587 cloning	pRGK368
MSP278	gcatagcctatgtatgtaggaatagagcctttttcatcaggg	pMCS587 cloning	pRGK368
MSP279	ccttatttacacagggcaggtgaataaccaatggctctacc	pMCS585 cloning	pRGK368
MSP280	ggtagagccattggttattcacctgccctgtgtaaataagg	pMCS585 cloning	pRGK368
